# Recombination in *Glomus intraradices*, a supposed ancient asexual arbuscular mycorrhizal fungus

**DOI:** 10.1186/1471-2148-9-13

**Published:** 2009-01-15

**Authors:** Daniel Croll, Ian R Sanders

**Affiliations:** 1 Department of Ecology & Evolution, Biophore building, University of Lausanne, CH-1015 Lausanne, Switzerland

## Abstract

**Background:**

Arbuscular mycorrhizal fungi (AMF) are important symbionts of most plant species, promoting plant diversity and productivity. This symbiosis is thought to have contributed to the early colonisation of land by plants. Morphological stasis over 400 million years and the lack of an observed sexual stage in any member of the phylum Glomeromycota led to the controversial suggestion of AMF being ancients asexuals. Evidence for recombination in AMF is contradictory.

**Results:**

We addressed the question of recombination in the AMF *Glomus intraradices *by sequencing 11 polymorphic nuclear loci in 40 morphologically identical isolates from one field. Phylogenetic relationships among genotypes showed a reticulate network pattern providing a rationale to test for recombination. Five statistical tests predicted multiple recombinant regions in the genome of a core set of isolates. In contrast, five clonal lineages had fixed a large number of differences.

**Conclusion:**

Our data show that AMF from one field have undergone recombination but that clonal lineages coexist. This finding has important consequences for understanding AMF evolution, co-evolution of AMF and plants and highlights the potential for commercially introduced AMF inoculum recombining with existing local populations. Finally, our results reconcile seemingly contradictory studies on whether AMF are clonal or form recombining populations.

## Background

Arbuscular mycorrhizal fungi (AMF) form symbioses with the majority of plants and influence their species diversity and productivity [1,2]. The symbiosis is thought to have existed ever since the colonization of land by plants [3]. Growth of AMF is thought to be entirely clonal by producing asexual spores and no sexual reproductive structures have been observed. Based on this fact, and the suggested 400 million years of morphological stasis [4], AMF were suggested to be ancient asexuals [5]. But over the past decade, the question whether AMF are asexuals or exhibit recombination has become a controversial issue [6,7]. Molecular evidence for recombination was previously found by analysis of the highly polymorphic *BiP *and rDNA sequences from *Glomus intraradices *and other AMF [8]. Recombination was detected among sequence variants present within single isolates. Due to the multigenomic nature of AMF, recombination could have been restricted to nuclei co-existing in the same cytoplasm, without recombination of DNA from genetically different individuals [9,10]. In populations of two related species, arbitrary genetic markers showed significant evidence for recombination [11]. Alternative explanations such as recurrent mutations or sequences from contaminating microorganisms could also explain these results because the AMF were not cultivated in clean culture prior to analysis. In contrast, two studies suggested a strict clonal evolution in populations through analysis of multiple polymorphic loci from spores of field populations [12,13]. In the first study, amplified fragment length polymorphism (AFLP) was scored for spores originating from a single pot of a cultured AMF isolate. Genetic diversity was found among spores but no evidence of recombination. The lack of replicate amplifications from single spores makes it difficult to reach a definite conclusion. Furthermore, the material originated for each species from a single pot culture and the fungi may not have had the opportunity to recombine with other genotypes from a field. In the second study, clonal reproduction in AMF was suggested by complete linkage of alleles at three loci among spores of field populations. Ideally, a much larger number of polymorphic loci should be investigated to draw conclusions about recombination. Using field-collected spores directly for genotyping would provide a more representative sample of the actual genetic diversity in an AMF population than using *in vitro *fungal cultures, as factors such as host plants used during cultivation could bias the composition of successfully established isolates [14]. However, currently only the *in vitro *system provides the required DNA quantities from fungi grown under sterile laboratory conditions necessary for reliable genotyping at a large number of loci [15]. Croll *et al*. [14] developed a set of 11 sequence-based markers to survey genetic diversity and host plant preferences in a population of 40 *G. intraradices *isolates established in an *in vitro *cultivation system. The genotyping was based on length polymorphism at nuclear and mitochondrial loci and sequencing of all loci in representative isolates was used to confirm locus specificity of the genotyping method. However, length polymorphism data alone are not suitable for recombination tests as length homoplasy of distinct sequences could introduce a strong bias. Using sequence information from all identified genotypes would allow a variety of tests for recombination and could, therefore, be used to challenge the fundamental assumption of ancient asexuality in AMF [16].

In strict terms, members of a morphospecies of unknown reproductive mode found together in the same location should not be called a population, as interbreeding of individuals is implied by the term population. For simplicity and in accordance with previous literature, we continue to use the term population to describe isolates of the same AMF species found in one location.

In this study, we use multi-locus sequence data of one *G. intraradices *population established in an *in vitro *system to (1) resolve phylogenetic relationships among genotypes that would indicate recombination or clonal evolution, and (2) use multiple sequence-based and population genetics methods to test for recombination in AMF. Detecting recombination in AMF would be important because it would further our understanding of a main fungal phylum, could have important consequences for understanding the co-evolution of AMF with plants and could have far reaching consequences for the use of commercial AMF inoculum.

## Results and Discussion

### Multi-locus genotypes of *G. intraradices *population

We used a set of 40 *in vitro *cultivated isolates of *G. intraradices *originating from one population in Tänikon, Switzerland, to address the question of occurrence of recombination in AMF. For reference, we included the isolate DAOM181602 originating from Québec, Canada. All isolates were clonally subcultured to obtain sufficient quantities of clean DNA [17]. Sequences of 11 polymorphic nuclear markers were used for a total alignment length of 3037 bp. These markers were initially developed to reveal sequence length polymorphisms and were identified using repeat-finding software. However, sequencing of the different alleles showed that the large majority of the detected polymorphism was found in regions flanking the repeat motifs. We found 75 polymorphic sites and 72 indel mutations (for details on polymorphism at each locus see additional file 1). The high degree of sequence polymorphism identified within the population corroborates earlier data on polymorphism in coding regions [18,19] and random genetic markers [17]. The distinct combinations of alleles at the 11 loci identified 17 genotypes in the population (labeled I–XI and XIII–XVIII). Isolate DAOM181602 was a unique genotype not found in the Swiss population (labeled XII). A sequence alignment of all 11 loci from the 18 genotypes is available as additional files 2 and 3. Identifying organisms by multi-locus sequence typing was pioneered by Maiden et al. [20] for bacteria and successfully applied to fungi to e.g. infer sources of human pathogen outbreaks [21].

### Phylogenetic relationships among genotypes

**Figure 1:**
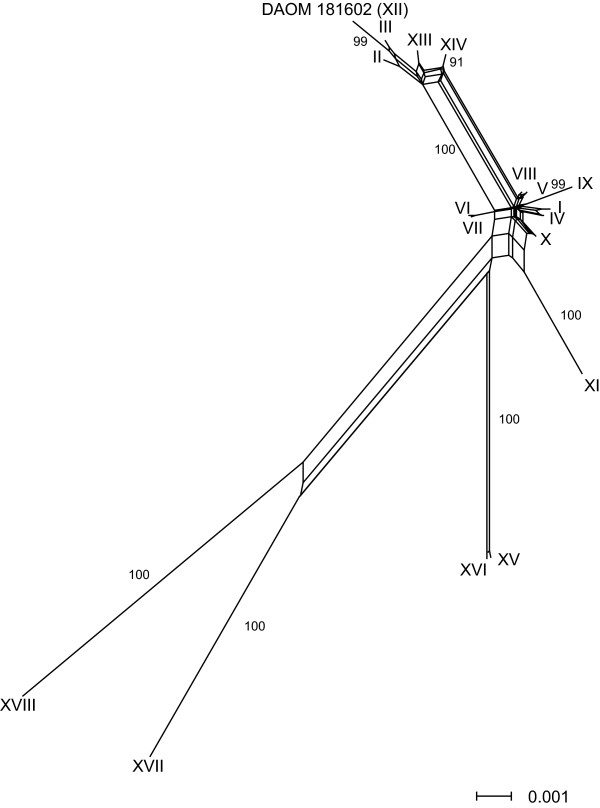
**Phylogenetic relationships among multi-locus genotypes of *G. intraradices***. The neighbour-network using uncorrected *p *distance showed reticulate phylogenetic branching among a core set of genotypes. Substitutions and indel mutations were given equal weight in the analysis. Bootstrap support for branches above 90% is indicated in % of 1000 replicates. Roman numerals represent the different genotypes from one field (I–XI and XIII–XVIIII) and DAOM181602 (XII).

We first estimated the phylogenetic relationships among the genotypes to identify potentially recombining genotypes and clonal lineages. Then, a series of statistical tests were performed on sequences from all genotypes to test whether there is significant evidence of recombination in the population. By applying a neighbour-network algorithm [22] reticulate paths connecting the core genotypes could be observed (I–X, DAOM181602, XIII, XIV; Fig. 1). A reticulate pattern suggests that recombination among genotypes may have contributed to the evolution of the genotypes, but multiple alternative mechanisms such as lack of phylogenetic resolution or homoplasy may also contribute to a reticulate pattern [23]. However, if recombination occurred within the population it is most likely among the core group of genotypes connected through a network (Fig. 1). The core genotypes weakly clustered into two subgroups, but multiple branches connect the two subgroups (I, IV–X and II, III, XIII, XIV, DAOM181602 respectively; Fig. 1). Five genotypes (XI, XV–XVIII) were distinguished by a comparatively large number of mutations from the set of core genotypes with very high bootstrap support (100%; Fig. 1). Three more genotypes were significantly differentiated from the core genotypes, but branch lengths were considerably shorter (IX, DAOM181602, XIV; Fig. 1). The reticulate pattern among the genotypes was conserved if all indel polymorphisms were excluded from the analysis (see additional file 4). This confirms that potential recurrent mutations, due to a higher indel mutation rate observed in repeat-rich regions, do not qualitatively alter the results of the analysis. The reticulate pattern among the core genotypes was still observed when the strongly differentiated genotypes (XI, XV–XVIII) were excluded, and this further supports the evidence of recombination (see additional file 4).

### Recombination detection in concatenated sequences

To examine evidence of recombination, we used multiple sequence-based tests, because detection abilities can vary strongly among tests depending on the degree of polymorphism and phylogenetic divergence of sequences [24,25]. We performed six tests of recombination on concatenated sequences of 11 loci. The concatenation of loci was necessary in order to overcome the limiting number of polymorphic sites within individual loci. Firstly, we used the Φ_w _test that has been shown to reliably discriminate between recurrent mutations and recombination [26]. The test uses a sliding-window procedure to assess phylogenetic compatibilities of nearby polymorphic sites in the concatenated sequences. A total of 72 sites were informative and significant evidence of recombination was found among the genotypes (*p *< 0.0001). We then used two phylogenetic (Bootscanning and RDP) and three nucleotide substitution based (Geneconv, MaxChi and Chimaera) tests [27-32]. These tests had already been applied to several fungal datasets in a critical analysis of statistical power of different recombination tests [24]. As sliding-window based tests are sensitive to the phylogenetic signals of neighbouring polymorphic sites, the concatenation order of our loci is likely to affect the detection abilities of the different tests. A pair of concatenated loci that would have been inherited clonally within the population is not expected to show evidence of recombination. However, for a pair of concatenated loci that show a reshuffling within the population, the tests are likely to provide evidence for recombination (i. e. a significant recombination breakpoint). As not all pairs of loci are expected to show similar levels of clonal inheritance or reshuffling, multiple concatenation orders are useful to control for a potential bias through the arbitrary nature of concatenation. In total, we used three different concatenation orders, two of which were random orders and the third separates loci with strong signals of recombination. We found that five isolates from the population (II, III, XI, XIII and XIV) and the commercially cultivated isolate DAOM181602 showed recombination breakpoints detected by two or more tests in all three concatenation orders. Furthermore, isolate XVIII showed recombination breakpoints by at least one test in all three concatenation orders. Isolate X did not show any recombination breakpoints in any of the concatenation orders. The graphical results showing significant recombination breakpoints are shown in additional files 5, 6, 7. Detailed results with significance thresholds for each predicted recombination breakpoint of the concatenation order shown in additional file 5 can be found in additional file 8. The presented recombination breakpoint analysis is not suitable to distinguish between intragenic recombination or reassortment of loci as sequences of individual loci are too short to perform the tests on each locus separately.

### Congruence analysis among loci

**Figure 2:**
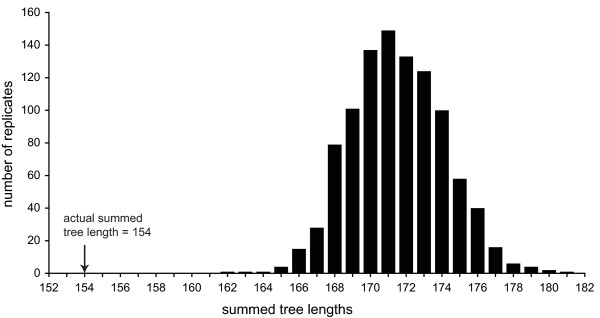
**Partition homogeneity test of 11 nuclear loci**. Partition homogeneity test showing the actual summed tree length by maximum parsimony of the dataset compared to summed tree lengths of 1000 artificially created datasets through re-sampling. The actual summed tree length is significantly shorter than the artificial datasets, indicating that incongruence exists among loci. Substitutions and indel mutations were given equal weight in the analysis.

Recombination creates conflicting phylogenies among distant loci through reshuffling of DNA sequences among different genomes. To test this prediction, we used a further, independent recombination test. The partition homogeneity test creates artificial datasets by sampling randomly among all observed sites of the genotypes and then swapping sites among loci [33,34]. The length of maximum parsimony trees for all loci are calculated and summed. If recombination occurred among the genotypes in the population, the actual summed tree length should be shorter than the summed tree lengths based on the artificially created datasets, because recombination should have introduced incongruence among loci. The partition homogeneity test showed that the actual summed tree length was 8 steps shorter than the shortest observed summed tree length in the artificially created dataset (*p *< 0.001, Fig. 2). Results were qualitatively similar if all indel polymorphisms were excluded (see additional file 9). Such conflicts in phylogenetic congruence among loci are most likely explained by recombination among the genotypes. The extent and frequency of recombination cannot, however, be inferred from these results. Results from the partition homogeneity test were shown to be sensitive to bias in base composition and mutation rate across loci [35,36], but as our study included individuals only from one population, we expect this influence to be negligible.

### Index of association among loci

The index of association measures the degree of linkage among different loci. This test has been widely used to detect linkage disequilibrium, following the initial application to bacterial genotypes [37]. In our study, we calculated the index of association for the complete set of genotypes as well as for the two different subsets of genotypes. According to the phylogenetic relationships among the 18 genotypes (Fig. 1), including all 18 genotypes in the analysis of index of association would be unlikely to reveal recombination because of a number of lineages that appear not to recombine with the core isolates. Indeed, analysis using all 18 genotypes revealed strong linkage disequilibrium (I_A _= 4.076, *p *= 0.0001), suggesting a population that deviates significantly from recombinant. If the five genotypes showing the strongest evidence for clonal evolution are excluded (see Fig. 1; bootstrap value = 100%; genotypes XI, XV–XVIII excluded) the linkage disequilibrium is no longer significant (I_A _= 0.226, *p *= 0.087). No linkage disequilibrium was detected either (I_A _= 0.167, *p *= 0.183) if all significantly separated genotypes are excluded (see Fig. 1; bootstrap value > 90%; genotypes IX, XI, XII, XIV–XVIII excluded), indicating that this subset of genotypes is potentially recombining. Two previous analyses of index of association in AMF populations showed opposite results from each other, although they studied different AMF species to those in the present study. Stukenbrock and Rosendahl [13] found strong linkage among three loci in three *Glomus *species, suggesting a strict clonal evolution. Vandenkoornhuyse, Leyval and Bonnin [11] found no evidence for linkage disequilibrium in two *Glomus *species based on ISSR loci. Our analysis showed that in a partially recombining AMF population, choosing various subsets of genotypes depending on the evidence of clonality changes the outcome of linkage analyses, but that groups of genotypes without significant linkage disequilibrium can indeed be detected.

### Evidence for recombination in an AMF population

Taken together, the graphical and statistical tests of recombination strongly suggest that recombination occurred among some of the genotypes in the field. The network analysis of the genotypes does not provide direct evidence for recombination, as other processes than recombination could lead to a reticulate pattern [22]. However, the analysis suggested that a core group of genotypes are the most likely candidates to have undergone recombination. The index of association analysis showed that, indeed, the strongest signal of recombination is among this core group of genotypes. The analysis of phylogenetic congruence among all genotypes using the partition homogeneity test strongly suggested the occurrence of recombination within the population. Furthermore, by combining the results of the six sequence-based tests using a sliding-window analysis, five isolates of the population showed significant recombination breakpoints supported by two or more tests in all three tested concatenations. Interestingly, the commercial inoculum DAOM181602 grouped into the core group of recombining *G. intraradices *isolates, with evidence supported by multiple tests. Recurrent mutations or homoplasy are unlikely to explain these results as excluding indel polymorphism and using robust methods do not qualitatively influence the results. A previous study of genetic diversity in the same population used length polymorphism data at the same loci as in this study to identify different isolates [14]. The reticulate relationships among the different genotypes suggested the occurrence of recombination as pointed out by Young [16]. However, complete sequencing of all loci in all genotypes and appropriate analyses would be required to reach a clear conclusion [16]. Homoplasy in the dataset would artificially increase signals of recombination as a pair of genotypes showing alleles of identical length but distinct sequence at a particular locus would be considered as potentially recombinant. The present study was based on sequences from all loci and all identified genotypes, therefore, allowing the control of size homoplasy. In future studies, data on genomic locations of the different loci could elucidate the frequency of recombination events and distinguish between inter- and intra-chromosomal recombination.

### Genetic heterogeneity in AMF

An alternative hypothesis could potentially explain patterns of recombination in sequences, as in some AMF it has been shown that isolates harbour genetically different nuclei [9,10]. The previous use of linkage disequilibrium analyses (i.e. index of association) on AMF spores using AFLP were shown to be misleading if only genetic variation within isolates was considered [10]. The sensitivity of fingerprinting techniques may artificially increase linkage disequilibrium and potentially mask recombination. Kuhn *et al*. [10] concluded that genetic heterogeneity found among nuclei within isolates has most likely arisen by accumulation of mutation, instead of recombination. To avoid the potentially confounding factor of within-isolate variation, we deliberately chose markers that did not reveal any significant within-isolate polymorphism (see Supporting Information in [14]). We have found other markers showing multiple alleles within single spore isolates. These were excluded from the analysis to rule out confounding within-isolate recombination but are the subject of further research. Thus, the genotypes included in our study describe the unique, or at least the overwhelmingly predominant, nuclear genotype present in each isolate. The evidence for this is that only single alleles were found at each locus within isolates using capillary electrophoresis (see Supporting Information in [14]). This argues against a potential explanation that is an alternative to recombination, namely that differences between isolates arise as a result of shifts in the relative frequency of pre-existing nuclear variants.

### Clonal evolution and potential for cryptic speciation

Our study reveals that a five isolates of *G. intraradices *isolates show strong evidence for recombination. However, we also identified five genotypes (XI, XV–XVIII) that were distinguished by a comparatively large number of independent mutational events, suggesting a clonal evolution in these lineages (Fig. 1). Statistical support for a clonal evolution of these genotypes was shown by very high bootstrap values separating these five lineages from the core group of genotypes (100%; Fig. 1). Several statistical tests identified putative recombination breakpoints in all these genotypes. However, these findings were not corroborated by different concatenation orders (additional files 5, 6, 7). This suggests that recombination between these latter genotypes and other genotypes in the population is either absent or rare. However, a much higher sampling effort in the field could yield genotypes that recombined with those lineages. Very few morphological criteria are known to distinguish isolates from the different clonal lineages, but the clonal lineages show clear differences in hyphal and spore densities produced in root organ cultures [17]. Furthermore, genetic differences among a subset of the currently studied AMF population were shown to affect symbiotic functions with host plants [38]. Knowledge of the effects on host plants by different clonal lineages and recombining genotypes would be important to understand the potential effects of recombination on AMF – host plant interactions. Recombination may have reduced genetic and phenotypic differentiation among the core genotypes, while clonal evolution in the separate lineages may have led to the fixation of a large number of genetic differences. In order to consider genotypes of different lineages as belonging to one biological species (i.e. forming an interbreeding population), all individuals should have the potential to undergo genetic exchange among each other. Our data, however, suggests that several lineages are independently evolving and may give rise to cryptic speciation.

### Detection of recombination in supposed ancient asexuals

The detection of recombination in a population of AMF is in strong contrast to the previous assumption of ancient asexuality in the phylum of Glomeromycota. The main argument for asexuality was based on morphological stasis over 400 million years, suggested by fossil evidence, and a lack of observed sexual structures [3,5]. Together with bdelloid rotifers and ostracods [5], AMF represent what Maynard Smith termed "evolutionary scandals" as accumulation of deleterious mutations and a slow rate of adaptation should strongly disadvantage asexual reproduction in comparison to sexuality in the long term [39]. While several theories tried to address this issue, recent evidence suggests that very few true asexuals might actually exist [8]. A more likely scenario is that genomes of most organisms undergo at least sporadic recombination events to cope with the deleterious effects of long term asexuality. Genomic signatures of recombination seem to be more pervasive than previously thought [8], highlighting the fundamental role of sex in the evolution of genomes.

### Sexual reproduction and vegetative incompatibility

Sexual reproductive cycles involving the fusion of genetically different hyphae (i.e. mating) are thought of being the main mechanism in fungi allowing recombination among different genomes [40]. But the lack of laboratory evidence of sexual structures involved in mating proved to be inconclusive about the occurrence of recombination in the species. Over the past decade, molecular evidence strongly suggested the occurrence of recombination in a number of fungal species that were presumed to strictly reproduce clonally. The human pathogen *Coccidioides immitis*, the pathogenic *Aspergillus flavus *and *A. fumigatus*, as well as the ant cultivated fungi in the fungus-growing ant symbiosis were all shown to undergo at least sporadic recombination events or even to possess functional genes required for meiosis [41-44]. In the case of *Cryptococcus neoformans*, where a known sexual cycle exists, population genetic structures were found to be almost completely clonal. Nevertheless, reproduction between individuals of the same mating type was shown to allow recombination to occur [45]. In a related species, a similar mechanism was shown to be at the origin of a major human pathogen outbreak [46].

In AMF, vegetative incompatibility was shown to occur when mycelia from different locations come into contact [47,48]. Such mechanisms, in combination with a lack of a known sexual cycle, were thought to exclude the possibility of recombination between genetically different isolates [49]. Our study suggests that these mechanisms need to be further researched within AMF populations as they are the most likely mechanisms allowing the mixing of nuclei and subsequent recombination among different AMF in the soil.

## Conclusion

Evidence for recombination in AMF has further implications than simply to remove these fungi from the list of putative ancient asexuals. Whether AMF are truly asexual or form recombining populations is critical to understanding AMF co-evolution with plants. Stability in mutualism can be favoured by asexuality of one partner, but this does not allow the symbiont to rapidly adapt to changes in the host [50]. Our study, therefore, has consequences for understanding the evolutionary potential of this mutualism. Different AMF genotypes in the population have different effects on plant growth [38]. Recombination among different genotypes may, therefore, produce AMF genotypes with novel effects on plant growth. Furthermore, evidence for recombination in AMF has an impact on commercially harnessing the beneficial traits of AMF for increasing crop production. The assumption of commercial AMF inoculum use is that an introduced AMF will not recombine with the local population. Our results highlight the potential that recombination could occur with the native AMF population and, by this, introduce new genes into the population. Finally, our findings reconcile previously published studies as recombination and clonality were both detected in AMF.

## Methods

### Origin of fungal isolates

A total of 40 *G. intraradices *isolates using in this study originated from an agricultural field site in Tänikon, Switzerland [51]. The same set of isolates was used for population genetic analyses using AFLP, simple sequence repeat and mitochondrial markers [14,17]. Species identity of all isolates was verified by Croll et al. [14] by sequencing the internal transcribed spacer region and subsequent comparison with deposited sequences of the same species. The *G. intraradices *isolate DAOM181602 originated from a field site at Pont Rouge, Quebec, Canada (Biosystematics Research Centre, Ottawa, Canada). *G. intraradices *is widely used as a commercial AMF inoculum for agricultural applications (Premier Tech Inc., Canada) and is the first AMF isolate to be entirely sequenced [52]. It is a haploid fungus with a compact genome of 15 Mb and is the only AMF for which ploidy has been measured [53]. Ploidy could be different for other AMF species [54].

### *In vitro *cultivation and DNA extraction

*In vitro *cultures of each isolate were established from single spores with Ri T-DNA-transformed carrot root on standard M growth medium [55]. Five to ten two-compartment plates were inoculated by clonal subculturing to allow the proliferation of the fungus in one compartment that is kept root-free, while remaining connected to the roots in the other compartment [17,56]. Root-free fungal compartments of all plates were pooled per single spore line for extraction of hyphae and spores. Freshly isolated hyphae and spores of each isolate were separately dried overnight at 48°C and ground into a fine powder using a Retsch MM300 mixer mill (Retsch, GmbH). The DNA was extracted using a modified version of the Cenis method for fungal DNA extraction with an additional step of 1:1 dilution with a solution of 24:1 of chloroform isoamyl alcohol before the final precipitation, to remove remaining impurities [57].

### PCR amplification of polymorphic loci

We applied tests of recombination on 18 different *G. intraradices *genotypes that were identified by Croll et al. [14] on the basis of variation at 11 nuclear loci. In the Swiss population, 17 genotypes were identified. The isolate DAOM181602 showed a unique genotype. Ten loci were shown to contain short regions of repetitive DNA, indel polymorphism and SNPs in the flanking regions of the repeats (see Supplementary Material in Croll et al. [14]). In addition, one nuclear gene intron was used [14]. The function of the gene is currently unknown. Each marker revealed only one allele per single spore isolate [14]. We identified other markers with multiple alleles per isolate but chose not to include these in the analysis to avoid confounding potential intra-isolate recombination with among isolate recombination (data unpublished). Croll et al. [14] identified alleles at each locus based on length differences and sequenced each allele that was seen to be different due to length polymorphism. For this study, we extended the sequencing, by sequencing all 11 loci in all the 18 genotypes identified by Croll et al. [14] so that any additional polymorphisms that were not based on length differences could be detected and also to check whether alleles with the same length in different isolates were also the same sequence. PCR amplifications were performed according to Croll et al. [14]. PCR products were purified using the MinElute PCR purification kit (Qiagen, Inc.). Purified and quantified PCR products were directly cycle sequenced with BigDye Terminator v1.1 (Applied Biosystems, Inc.) following the supplier's instructions. Cycle sequence products were purified by ethanol precipitation. An ABI PRISM™ 3100 Genetic Analyzer was used for automated sequencing. All sequence profiles were visually checked using 4 peaks software (A. Griekspoor and T. Groothuis, ). Sequences of all loci were aligned using the ClustalW algorithm implemented CLCbio Free Workbench 4.0 software  and alignments were checked manually. Independent PCR and re-sequencing of alleles was performed to check for potential genotyping errors for all loci and in several isolates. No sequence variation was found in these tests. Identical labels (roman numerals I–XVIII) were used to name the genotypes as in the previous study [14]. The sequences at all 11 loci of the 18 genotypes were deposited: locus Bg32 [GenBank: EU534209–26]; Bg42 [GenBank: EU534227–44]; Bg62 [GenBank: EU534245–62]; Bg196 [GenBank: EU534263–80]; Bg235 [GenBank: EU534281–98]; Bg273 [GenBank: EU534299–316]; Bg276 [GenBank: EU534316–34]; Bg303 [GenBank: EU534335–52]; Bg348 [GenBank: EU534353–70]; Bg355 [GenBank: EU534371–88]; nuclear intron [GenBank: EU534389–406]. A sequence alignment of all genotypes with concatenated loci as used for the data analyses is available in additional files 2 and 3.

### Phylogenetic analysis and tests for recombination

The neighbour-net algorithm implemented in SplitsTree 4.8 was used to construct a network based on the uncorrected *p *distance using concatenated sequences of all loci [22]. The robustness of the branching pattern was assessed with 1000 bootstrap replicates. Analyses were performed in two ways: (1) coding indel and repeat polymorphisms with Gapcoder using 1/0 to code for presence or absence of gaps in the alignment [58] and (2) ignoring all indel polymorphisms (see additional file 4). Ignoring gaps reduces the risk of including polymorphisms that may be due to recurrent mutations occurring in short repeat motifs, confounding potential signatures of recombination. Nevertheless, the majority of indel mutations are not in repetitive stretches of DNA and are, therefore, unlikely to be recurrent mutations.

Following Posada [24] and Posada and Crandall [30], we applied several recombination tests to evaluate the robustness of our results. To test for evidence of recombination based on phylogenetic compatibilities of nearby polymorphic sites along concatenated sequences, the Φ_w _test implemented in Splitstree 4.8 was used [26,59]. The test was run with the default settings of a window size of 100 and k = 2. Five additional recombination tests were run in order to predict putative recombinant regions in the concatenated sequences: (1) Geneconv [29,32] was performed scanning sequence triplets and treating indel blocks as single polymorphisms. (2) MaxChi [28] was used to scan all possible sequence triplets with 30 variable sites per window, alignments gaps (indels) were not considered as this could generate false positives [27]. (3) General recombination detection (RDP test implemented in RDP software; [27]) was performed with a window size of 10 and without specifying a reference sequence. (4) Bootscanning [31] was used with a window size of 100 and a step size of 20, standard distances were used for calculations and *p *values were binomial. (5) Chimaera [30] is a modification of the MaxChi test and was run with 30 variable sites per window. All five tests were run with RDP v2.08 [27], setting the significance threshold to *p *= 0.05. Multiple comparison corrections were performed by a Bonferroni correction [27].

### Partition homogeneity test

The partition homogeneity test [33,34] implemented in PAUP 4.0b10 [60] was used to estimate the degree of incongruence among loci created by recombination. To compare the degree of incongruence, 1000 datasets were artificially created by re-sampling all sites without replacement, regardless of the loci boundaries. The summed tree length of the maximum parsimony trees of each locus found for the original dataset was compared to the summed tree lengths of the maximum parsimony trees of each locus for the artificially created dataset. Firstly, the analysis was performed giving equal weight to substitutions and indel polymorphisms, giving a total of 77 parsimony-informative sites. An additional 70 variable sites were parsimony-uninformative. Secondly, the analysis was performed considering only substitutions in the sequences. This provided 32 parsimony-informative characters and 43 parsimony-uninformative characters (for results see additional file 9).

### Index of association

The index of association (I_A_) measures the degree of linkage equilibrium among a set of genotypes. I_A _= V_O_/V_E _-1, where V_O _is the observed variance of the number of loci being different within all pairs of genotypes and V_E _is the variance of the number of loci being different within all pairs of genotypes expected under complete linkage equilibrium. Therefore, I_A _= 0 if genotypes recombine freely. The I_A _and the significance threshold of a deviation from linkage equilibrium (with 10'000 randomizations) were calculated with the program MultiLocus v1.3b developed by P.-M. Agapow and A. Burt [61].

## Authors' contributions

DC conceived the study, performed the molecular and statistical analyses and drafted the manuscript. IRS participated in the design of the study and helped to draft the manuscript.

## Supplementary Material

Additional file 1 **Polymorphism found in sequences of 11 loci among 17 genotypes of Glomus intraradices from one field and the isolate DAOM181602.** Click here for file

Additional file 2 **Sequence alignment of 11 concatenated loci of 18 genotypes of *G. intradices*. Different shades indicate different loci.** Click here for file

Additional file 3 **Sequence alignment of 11 concatenated loci of 18 genotypes of *G. intraradices *in fasta sequence format.** Click here for file

Additional file 4 **Phylogenetic relationships among genotypes in a population of *G. intraradices *based on a Neighbour-network using uncorrected *p *distance.** Click here for file

Additional file 5 **Summary of five recombination tests based on the concatenated sequences of 11 nuclear loci.** Loci are concatenated arbitrarily according to the locus labelling. For individual tests, recombinant regions can overlap as all significant recombinant regions were kept in the analysis. Significance was based on *p *< 0.05, corrected for multiple comparisons. For exact *p *values for all putative recombinant regions see additional file 2. The total alignment length is 3037 bp. Click here for file

Additional file 6 **Summary of five recombination tests based on the concatenated sequences of 11 nuclear loci.** Loci Bg62, Bg196 and Bg235 showing strong signals of recombination in additional file 5 are not consecutive. Click here for file

Additional file 7 **Summary of five recombination tests based on the concatenated sequences of 11 nuclear loci**. Loci are concatenated in a 2^nd ^arbitrary order compared to additional file 5. Click here for file

Additional file 8 **Details of the recombination breakpoint analysis with the Recombination Detection Program on concatenated sequences of the 11 loci among 17 genotypes of one field of *Glomus intraradices *and the isolate DAOM181602**. The concatenation order corresponds to the one presented in additional file 5. Click here for file

Additional file 9 **Partition homogeneity test of 11 nuclear loci with all indels being removed.** Click here for file

## References

[B1] Smith SE, Read DJ (1997). Mycorrhizal Symbiosis.

[B2] Heijden MGA Van der, Klironomos JN, Ursic M, Moutoglis P, Streitwolf-Engel R, Boller T, Wiemken A, Sanders IR (1998). Mycorrhizal fungal diversity determines plant biodiversity, ecosystem variability and productivity. Nature.

[B3] Redecker D, Kodner R, Graham LE (2000). Glomalean fungi from the Ordovician. Science.

[B4] Remy W, Taylor TN, Hass H, Kerp H (1994). Four hundred-million-year-old vesicular arbuscular mycorrhizae. Proc Natl Acad Sci USA.

[B5] Judson OP, Normark BB (1996). Ancient asexual scandals. Trends Ecol Evol.

[B6] Pawlowska TE, Heitman J, Kronstad JW, Taylor JW, Casselton LA (2007). How the genome is organized in the Glomeromycota. Sex in Fungi: Molecular Determination and Evolutionary Implications.

[B7] Rosendahl S (2008). Communities, populations and individuals of arbuscular mycorrhizal fungi. New Phyt.

[B8] Gandolfi A, Sanders IR, Rossi V, Menozzi P (2003). Evidence of recombination in putative ancient asexuals. Mol Biol Evol.

[B9] Hijri M, Sanders IR (2005). Low gene copy number shows that arbuscular mycorrhizal fungi inherit genetically different nuclei. Nature.

[B10] Kuhn G, Hijri M, Sanders IR (2001). Evidence for the evolution of multiple genomes in arbuscular mycorrhizal fungi. Nature.

[B11] Vandenkoornhuyse P, Leyval C, Bonnin I (2001). High genetic diversity in arbuscular mycorrhizal fungi: evidence for recombination events. Heredity.

[B12] Rosendahl S, Taylor JW (1997). Development of multiple genetic markers for studies of genetic variation in arbuscular mycorrhizal fungi using AFLP. Mol Ecol.

[B13] Stukenbrock EH, Rosendahl S (2005). Clonal diversity and population genetic structure of arbuscular mycorrhizal fungi (*Glomus *spp.) studied by multilocus genotyping of single spores. Mol Ecol.

[B14] Croll D, Wille L, Gamper HA, Mathimaran N, Lammers PJ, Corradi N, Sanders IR (2008). Genetic diversity and host plant preferences revealed by simple sequence repeat and mitochondrial markers in a population of the arbuscular mycorrhizal fungus Glomus intraradices. New Phytologist.

[B15] Croll D, Corradi N, Gamper HA, Sanders IR (2008). Multilocus genotyping of arbuscular mycorrhizal fungi and marker suitability for population genetics. New Phyt.

[B16] Young JP (2008). The genetic diversity of intraterrestrial aliens. New Phytol.

[B17] Koch AM, Kuhn G, Fontanillas P, Fumagalli L, Goudet I, Sanders IR (2004). High genetic variability and low local diversity in a population of arbuscular mycorrhizal fungi. Proc Natl Acad Sci USA.

[B18] Corradi N, Croll D, Colard A, Kuhn G, Ehinger M, Sanders IR (2007). Gene copy number polymorphisms in an arbuscular mycorrhizal fungal population. Appl Env Microb.

[B19] Corradi N, Sanders IR (2006). Evolution of the P-type II ATPase gene family in the fungi and presence of structural genomic changes among isolates of *Glomus intraradices*. BMC Evol Biol.

[B20] Maiden MCJ, Bygraves JA, Feil E, Morelli G, Russell JE, Urwin R, Zhang Q, Zhou JJ, Zurth K, Caugant DA (1998). Multilocus sequence typing: A portable approach to the identification of clones within populations of pathogenic microorganisms. Proc Natl Acad Sci USA.

[B21] Fisher MC, Rannala B, Chaturvedi V, Taylor JW (2002). Disease surveillance in recombining pathogens: Multilocus genotypes identify sources of human Coccidioides infections. Proc Natl Acad Sci USA.

[B22] Huson DH, Bryant D (2006). Application of phylogenetic networks in evolutionary studies. Mol Biol Evol.

[B23] Bryant D, Moulton V (2004). Neighbor-Net: An agglomerative method for the construction of phylogenetic networks. Mol Biol Evol.

[B24] Posada D (2002). Evaluation of methods for detecting recombination from DNA sequences: Empirical data. Mol Biol Evol.

[B25] Posada D, Crandall KA, Holmes EC (2002). Recombination in evolutionary genomics. Annu Rev Gen.

[B26] Bruen TC, Philippe H, Bryant D (2006). A simple and robust statistical test for detecting the presence of recombination. Genetics.

[B27] Martin D, Rybicki E (2000). RDP: detection of recombination amongst aligned sequences. Bioinformatics.

[B28] Maynard Smith JM (1992). Analyzing the mosaic structure of genes. J Mol Evol.

[B29] Padidam M, Sawyer S, Fauquet CM (1999). Possible emergence of new geminiviruses by frequent recombination. Virology.

[B30] Posada D, Crandall KA (2001). Evaluation of methods for detecting recombination from DNA sequences: Computer simulations. Proc Natl Acad Sci USA.

[B31] Salminen MO, Carr JK, Burke DS, McCutchan FE (1995). Identification of breakpoints in intergenotypic recombinants of HIV type 1 by bootscanning. AIDS research and human retroviruses.

[B32] Sawyer S (1989). Statistical Tests for Detecting Gene Conversion. Mol Biol Evol.

[B33] Farris JS, Kallersjo M, Kluge AG, Bult C (1994). Testing Significance of Incongruence. Cladistics.

[B34] Farris JS, Kallersjo M, Kluge AG, Bult C (1995). Constructing a significance test for incongruence. Syst Biol.

[B35] Hipp AL, Hall JC, Sytsma KJ (2004). Congruence versus phylogenetic accuracy: Revisiting the incongruence length difference test. Syst Biol.

[B36] Lee MSY (2001). Uninformative characters and apparent conflict between molecules and morphology. Mol Biol Evol.

[B37] Maynard Smith J, Smith NH, Orourke M, Spratt BG (1993). How Clonal Are Bacteria?. Proc Natl Acad Sci USA.

[B38] Koch AM, Croll D, Sanders IR (2006). Genetic variability in a population of arbuscular mycorrhizal fungi causes variation in plant growth. Ecol Lett.

[B39] Maynard Smith J (1986). Evolution – Contemplating Life without Sex. Nature.

[B40] Kronstad JW, Staben C (1997). Mating type in filamentous fungi. Annu Rev Gen.

[B41] Burt A, Carter DA, Koenig GL, White TJ, Taylor JW (1996). Molecular markers reveal cryptic sex in the human pathogen Coccidioides immitis. Proc Natl Acad Sci USA.

[B42] Geiser DM, Pitt JI, Taylor JW (1998). Cryptic speciation and recombination in the aflatoxin-producing fungus Aspergillus flavus. Proc Natl Acad Sci USA.

[B43] Mikheyev AS, Mueller UG, Abbot P (2006). Cryptic sex and many-to-one colevolution in the fungus-growing ant symbiosis. Proc Natl Acad Sci USA.

[B44] Paoletti M, Rydholm C, Schwier EU, Anderson MJ, Szakacs G, Lutzoni F, Debeaupuis JP, Latge JP, Denning DW, Dyer PS (2005). Evidence for sexuality in the opportunistic fungal pathogen Aspergillus fumigatus. Current Biology.

[B45] Lin X, Hull CM, Heitman J (2005). Sexual reproduction between partners of the same mating type in Cryptococcus neoformans. Nature.

[B46] Fraser JA, Giles SS, Wenink EC, Geunes-Boyer SG, Wright JR, Diezmann S, Allen A, Stajich JE, Dietrich FS, Perfect JR (2005). Same-sex mating and the origin of the Vancouver Island Cryptococcus gattii outbreak. Nature.

[B47] Giovannetti M, Azzolini D, Citernesi AS (1999). Anastomosis formation and nuclear and protoplasmic exchange in arbuscular mycorrhizal fungi. Appl Env Microb.

[B48] Giovannetti M, Sbrana C, Strani P, Agnolucci M, Rinaudo V, Avio L (2003). Genetic diversity of isolates of *Glomus mosseae *from different geographic areas detected by vegetative compatibility testing and biochemical and molecular analysis. Appl Env Microb.

[B49] Pawlowska TE, Taylor JW (2005). Arbuscular mycorrhizal fungi: Hyphal fusion and multigenomic structure (reply). Nature.

[B50] Law R, Boucher DH (1985). Evolution in a mutualistic environment. The biology of mutualism.

[B51] Jansa J, Mozafar A, Anken T, Ruh R, Sanders IR, Frossard E (2002). Diversity and structure of AMF communities as affected by tillage in a temperate soil. Mycorrhiza.

[B52] Lammers P, Tuskan GA, DiFazio SP, Podila GK, Martin F (2004). Mycorrhizal symbionts of *Populus *to be sequenced by the United States Department of Energy's Joint Genome Institute. Mycorrhiza.

[B53] Hijri M, Sanders IR (2004). The arbuscular mycorrhizal fungus *Glomus intraradices *is haploid and has a small genome size in the lower limit of eukaryotes. Fungal Gen Biol.

[B54] Pawlowska TE, Taylor JW (2004). Organization of genetic variation in individuals of arbuscular mycorrhizal fungi. Nature.

[B55] Bécard G, Fortin JA (1988). Early events of vesicular arbuscular mycorrhiza formation on Ri T-DNA transformed roots. New Phyt.

[B56] St.-Arnaud M, Hamel C, Vimard B, Caron M, Fortin JA (1996). Enhanced hyphal growth and spore production of the arbuscular mycorrhizal fungus *Glomus intraradices *in an *in vitro *system in the absence of host roots. Mycol Res.

[B57] Cenis JL (1992). Rapid extraction of fungal DNA for PCR amplification. Nucleic Acids Res.

[B58] Young ND, Healy J (2003). GapCoder automates the use of indel characters in phylogenetic analysis. BMC Bioinformatics.

[B59] Dress A, Huson D, Moulton V (1996). Analyzing and visualizing sequence and distance data using SPLITSTREE. Discrete Appl Math.

[B60] Swofford DL (2002). PAUP*: Phylogenetic Analysis Using Parsimony, Version 4b10.

[B61] MultiLocus Software. http://www.bio.ic.ac.uk/evolve/software/multilocus.

