# Supplementary material for: Recombination in Glomus intraradices, a supposed ancient asexual arbuscular mycorrhizal fungus
Source: BMC Evol Biol. 2009 Jan 15;9:13. doi: 10.1186/1471-2148-9-13 (PMC2630297; doi:10.1186/1471-2148-9-13)
Supplement: Additional file 1 — Polymorphism found in sequences of 11 loci among 17 genotypes of Glomus intraradices from one field and the isolate DAOM181602. [file 1471-2148-9-13-S1.pdf]

Additional file 1 – Polymorphism found in sequences of 11 loci among 17 genotypes of Glomus intraradices from one field and the isolate DAOM181602.

Loci	Aligned length (bp)	Substitutions	Indel
Bg32	273	18	6
Bg42	274	7	5
Bg62	315	0	4
Bg196	315	11	8
Bg235	331	10	6
Bg273	253	5	7
Bg276	314	6	6
Bg303	136	0	2
Bg348	214	0	7
Bg355	332	11	3
nr intron*	280	7	18
Total	3037	75	72

* the nuclear intron was identified by Croll et al. (2008) in an EST of currently unknown function.
