# Supplementary material for: Recombination in Glomus intraradices, a supposed ancient asexual arbuscular mycorrhizal fungus
Source: BMC Evol Biol. 2009 Jan 15;9:13. doi: 10.1186/1471-2148-9-13 (PMC2630297; doi:10.1186/1471-2148-9-13)
Supplement: Additional file 2 — Sequence alignment of 11 concatenated loci of 18 genotypes of G. intradices. Different shades indicate different loci. [file 1471-2148-9-13-S2.pdf]

Additional file 5 - Alignment of 11 nuclear loci of 18 genotypes found in *G. intraradices*.

```
                                                                                  Alignment
Genotypes     Loci (shaded in black/grey)                                          position
              Bg32                                                                       60
I             CAACCATTGGCTTCAGAAACAGCATTATAATCTCTAAGCCAAGCAACAGGATCTTGCTGA
II            CAACCATTGGCTTCAGAAACAGCATTATAATCTCTAAGCCAAGCAACAGGATCTTGCTGA
III           CAACCATTGGCTTCAGAAACAGCATTATAATCTCTAAGCCAAGCAACAGGATCTTGCTGA
IV            CAACCATTGGCTTCAGAAACAGCATTATAATCTCTAAGCCAAGCAACAGGATCTTGCTGA
V             CAACCATTGGCTTCAGAAACAGCATTATAATCTCTAAGCCAAGCAACAGGATCTTGCTGA
VI            CAACCATTGGCTTCAGAAACAGCATTATAATCTCTAAGCCAAGCAACAGGATCTTGCTGA
VII           CAACCATTGGCTTCAGAAACAGCATTATAATCTCTAAGCCAAGCAACAGGATCTTGCTGA
VIII          CAACCATTGGCTTCAGAAACAGCATTATAATCTCTAAGCCAAGCAACAGGATCTTGCTGA
IX            CAACCATTGGCTTCAGAAACAGCATTATAATCTCTAAGCCAAGCAACAGGATCTTGCTGA
X             CAACCATTGGCTTCAGAAACAGCATTATAATCTCTAAGCCAAGCAACAGGATCTTGCTGA
XI            CAACCATTGGCTTCAGAAACAGCATTATAATCTCTAAGCCAAGCAACAGGATCTTACTGA
XII           CAACCATTGGCTTCAGAAACAGCATTATAATCTCTAAGCCAAGCAACAGGATCTTGCTGA
XIII          CAACCATTGGCTTCAGAAACAGCATTATAATCTCTAAGCCAAGCAACAGGATCTTGCTGA
XIV           CAACCATTGGCTTCAGAAACAGCATTATAATCTCTAAGCCAAGCAACAGGATCTTGCTGA
XV            CAACCATTGGCTTCAGAAACAGCATTATAATCTCTAAGCCAAGCAACAGGATCTTGCTGA
XVI           CAACCATTGGCTTCAGAAACAGCATTATAATCTCTAAGCCAAGCAACAGGATCTTGCTGA
XVII          CAACCATTGGCTTCAGAAACAGCATTATAATCTCTAAGCCAAGCAACAGGATCTTGCTGA
XVIII         CAACCATTGGCTTCAGAAACAGCATTATAATCTCTAAGCCAAGCAACAGGATCTTGCTGA
Conservation  ******************************************************* ****

              Bg32                                                                      120
I             TTTTCTCCACTAAAAGTTGGTAAAAATGCTCCCCAACTTTGCTGATTCTGCTGGTTTTGT
II            TTTTCTCCACTAAAAGTTGGTAAAAATGCTCCCCAACTTTGCTGATTCTGCTGGTTTTGT
III           TTTTCTCCACTAAAAGTTGGTAAAAATGCTCCCCAACTTTGCTGATTCTGCTGGTTTTGT
IV            TTTTCTCCACTAAAAGTTGGTAAAAATGCTCCCCAACTTTGCTGATTCTGCTGGTTTTGT
V             TTTTCTCCACTAAAAGTTGGTAAAAATGCTCCCCAACTTTGCTGATTCTGCTGGTTTTGT
VI            TTTTCTCCACTAAAAGTTGGTAAAAATGCTCCCCAACTTTGCTGATTCTGCTGGTTTTGT
VII           TTTTCTCCACTAAAAGTTGGTAAAAATGCTCCCCAACTTTGCTGATTCTGCTGGTTTTGT
VIII          TTTTCTCCACTAAAAGTTGGTAAAAATGCTCCCCAACTTTGCTGATTCTGCTGGTTTTGT
IX            TTTTCTCCACTAAAAGTTGGTAAAAATGCTCCCCAACTTTGCTGATTCTGCTGGTTTTGT
X             TTTTCTCCACTAAAAGTTGGTAAAAATGCTCCCCAACTTTGCTGATTCTGCTGGTTTTGT
XI            TTTTCTCCACTAAAAGTTGGTAAGAACGCTCCCCAACTTTGCTGATTCTGCTGGGTTTGT
XII           TTTTCTCCACTAAAAGTTGGTAAAAATGCTCCCCAACTTTGCTGATTCTGCTGGTTTTGT
XIII          TTTTCTCCACTAAAAGTTGGTAAAAATGCTCCCCAACTTTGCTGATTCTGCTGGTTTTGT
XIV           TTTTCTCCACTAAAAGTTGGTAAAAATGCTCCCCAACTTTGCTGATTCTGCTGGTTTTGT
XV            TTTTCTCCACTAAAAGTTGGTAAGA-TGCGCCCCAACTTTGCTGATTCTGCTGGTTTTGT
XVI           TTTTCTCCACTAAAAGTTGGTAAGA-TGCGCCCCAACTTTGCTGATTCTGCTGGTTTTGT
XVII          TTTTCTCCACTAAAAGTTGGCAAGAATGCTCCCCAACTTTGCTGATTCTGCTGGTTTTGT
XVIII         TTTTCTCCACTAAAAGTTGGTAAAAATGCTCCCCAACTTTGCTGATTCTGCTGGTTTTGT
Conservation  ******************** ** *  ** ************************ *****

              Bg32                                                                      180
I             TGATTTAAAA-TTGTTGG-----------ATGAACTGGGCCATCTGCAACATATTATTTC
II            TGATTTAAAAATTGTTGG-----------ATGAACTGGGCCATCTGCAACATATTATTTC
III           TGATTTAAAA-TTGTTGG-----------ATGAACTGGGCCATCTGCAACATATTATTTC
IV            TGATTTAAAA-TTGTTGG-----------ATGAACTGGGCCATCTGCAACATATTATTTC
V             TGATTTAAAA-TTGTTGG-----------ATGAACTGGGCCATCTGCAACATATTATTTC
VI            TGATTTAAAA-TTGTTGG-----------ATGAACTGGGCCATCTGCAACATATTATTTC
VII           TGATTTAAAAATTGTTGG-----------ATGAACTGGGCCATCTGCAACATATTATTTC
VIII          TGATTTAAAAATTGTTGG-----------ATGAACTGGGCCATCTGCAACATATTATTTC
IX            TGATTTAAAA-TTGTTGG-----------ATGAACTGGGCCATCTGCAACATATTATTTC
X             TGATTTAAAA-TTGTTGG-----------ATGAACTGGGCCATCTGCAACATATTATTTC
XI            TGATTTAAAAATTGTTGGG----------ATGAACTGGGCCATCTGCAACATATTATTTC
XII           TGATTTAAAAATTGTTGG-----------ATGAACTGGGCCATCTGCAACATATTATTTC
XIII          TGATTTAAAAATTGTTGG-----------ATGAACTGGGCCATCTGCAACATATTATTTC
XIV           TGATTTAAAA-TTGTTGG-----------ATGAACTGGGCCATCTGCAACATATTATTTC
XV            TGATTTAAAAATTGTTGGG----------AGGAACTGGGCGATCTGCAACATATTATTTC
XVI           TGATTTAAAAATTGTTGGG----------AGGAACTGGGCGATCTGCAACATATTATTTC
XVII          TGATTTAAAAATTGTTGGATAAACTGGGAGTAAACTGGGCCATCTGCAACATATTATTTC
XVIII         TGATTTAAAAATTGTTGG-----------ATGAACTGGGCCATCTGCAACATATTATTTC
Conservation  ********** *******              ******** *******************

              Bg32                                                                      240
I             TTTCCAAAAAGTTACCATTATTAACATTGTCATTCTGATTGTTGTTATTATCTCATGGAA
II            TTTCCAAAAAGTTACCATTATTAACATTGTCATTCTGATTGTTGTTATTATCTCATGGAA
III           TTTCCAAAAAGTTACCATTATTAACATTGTCATTCTGATTGTTGTTATTATCTCATGGAA
IV            TTTCCAAAAAGTTACCATTATTAACATTGTCATTCTGATTGTTGTTATTATCTCATGGAA
V             TTTCCAAAAAGTTACCATTATTAACATTGTCATTCTGATTGTTGTTATTATCTCATGGAA
VI            TTTCCAAAAAGTTACCATTATTAACATTGTCATTCTGATTGTTGTTATTATCTCATGGAA
VII           TTTCCAAAAAGTTACCATTATTAACATTGTCATTCTGATTGTTGTTATTATCTCATGGAA
VIII          TTTCCAAAAAGTTACCATTATTAACATTGTCATTCTGATTGTTGTTATTATCTCATGGAA
IX            TTTCCAAAAAGTTACCATTATTAACATTGTCATTCTGATTGTTGTTATTATCTCATGGAA
X             TTTCCAAAAAGTTACCATTATTAACATTGTCATTCTGATTGTTGTTATTATCTCATGGAA
XI            CTTCCAAAAAGTTACCATTATTAACATTGTTATTCTGATTGTTGCTATTATTTCGTGAAA
XII           TTTCCAAAAAGTTACCATTATTAACATTGTCATTCTGATTGTTGTTATTATCTCATGGAA
XIII          TTTCCAAAAAGTTACCATTATTAACATTGTCATTCTGATTGTTGTTATTATCTCATGGAA
XIV           TTTCCAAAAAGTTACCATTATTAACATTGTCATTCTGATTGTTGTTATTATCTCATGGAA
XV            TTTCCAAAAAGTTACCATTTT---------CATTCTGATTGTTGTT----------GGAA
XVI           TTTCCAAAAAGTTACCATTTT---------CATTCTGATTGTTGTT----------GGAA
XVII          CTTCCAAAAAGTTACCATTATTAATATTATTATTCTGATTGTTGTTATTATCTCATGGAA
XVIII         TTTCCAAAAAGTTACCATTATTAACATTGTCATTCTGATTGTTGTTATTATCTCATGGAA
Conservation   ****************** *          ************* *          * **
```

```
Bg32                                                                        300
I             CTGGATCTCAAACTTGTTGCTGAAACAATCAAACTCGCTAAAATTTGCCGATCTTTTTTA
II            CTGGATCTCAAACTTGTTGCTGAAACAATCAAACTCGCTAAAATTTGCCGATCTTTTTTA
III           CTGGATCTCAAACTTGTTGCTGAAACAATCAAACTCGCTAAAATTTGCCGATCTTTTTTA
IV            CTGGATCTCAAACTTGTTGCTGAAACAATCAAACTCGCTAAAATTTGCCGATCTTTTTTA
V             CTGGATCTCAAACTTGTTGCTGAAACAATCAAACTCGCTAAAATTTGCCGATCTTTTTTA
VI            CTGGATCTCAAACTTGTTGCTGAAACAATCAAACTCGCTAAAATTTGCCGATCTTTTTTA
VII           CTGGATCTCAAACTTGTTGCTGAAACAATCAAACTCGCTAAAATTTGCCGATCTTTTTTA
VIII          CTGGATCTCAAACTTGTTGCTGAAACAATCAAACTCGCTAAAATTTGCCGATCTTTTTTA
IX            CTGGATCTCAAACTTGTTGCTGAAACAATCAAACTCGCTAAAATTTGCCGATCTTTTTTA
X             CTGGATCTCAAACTTGTTGCTGAAACAATCAAACTCGCTAAAATTTGCCGATCTTTTTTA
XI            CTGGATCTCAAACTTGTTGCTGAAACAATCAAACTCGCTAAAATTTGCCGATCTTTTTTA
XII           CTGGATCTCAAACTTGTTGCTGAAACAATCAAACTCGCTAAAATTTGCCGATCTTTTTTA
XIII          CTGGATCTCAAACTTGTTGCTGAAACAATCAAACTCGCTAAAATTTGCCGATCTTTTTTA
XIV           CTGGATCTCAAACTTGTTGCTGAAACAATCAAACTCGCTAAAATTTGCCGATCTTTTTTA
XV            CTGGATCTCAAACTTGTTGCTGAAACAATCAAACTCGCTAAAATTTGCCGATCTTTTTTA
XVI           CTGGATCTCAAACTTGTTGCTGAAACAATCAAACTCGCTAAAATTTGCCGATCTTTTTTA
XVII          CTGGATCTCAAACTTGTTGCTGAAACAATCAAACTCGCTAAAATTTGCCGATCTTTTTTA
XVIII         CTGGATCTCAAACTTGTTGCTGAAACAATCAAACTCGCTAAAATTTGCCGATCTTTTTTA
Conservation  ************************************************************

Bg42                                                                        360
I             TTTTTACTATTTTTACATAAACTTTATTCGTTTTATTTATTATATATATGAATATATA--
II            TTTTTACTATTTTTACATAAACTTTATTCGTTTTATTTATTATATATATGAATATATA--
III           TTTTTACTATTTTTACATAAACTTTATTCGTTTTATTTATTATATATATGAATATATA--
IV            TTTTTACTATTTTTACATAAACTTTATTCGTTTTATTTATTATATATATGAATATATA--
V             TTTTTACTATTTTTACATAAACTTTATTCGTTTTATTTATTATATATATGAATATATA--
VI            TTTTTACTATTTTTACATAAACTTTATTCGTTTTATTTATTATATATATGAATATATA--
VII           TTTTTACTATTTTTACATAAACTTTATTCGTTTTATTTATTATATATATGAATATATA--
VIII          TTTTTACTATTTTTACATAAACTTTATTCGTTTTATTTATTATATATATGAATATATA--
IX            TTTTTACTATTTTTACATAAACTTTATTCGTTTTATTTATTATATATATGAATATATA--
X             TTTTTACTATTTTTACATAAACTTTATTCGTTTTATTTATTATATATATGAATATATA--
XI            TTTTTACTATTTTTACATAAACTTTATTCGTTTTATTTATTATATATATGAATATATA--
XII           TTTTTACTATTTTTACATAAACTTTATTCGTTTTATTTATTATATATATGAATATATA--
XIII          TTTTTACTATTTTTACATAAACTTTATTCGTTTTATTTATTATATATATGAATATATA--
XIV           TTTTTACTATTTTTACATAAACTTTATTCGTTTTATTTATTATATATATGAATATATA--
XV            TTTTTACTATTTTTACATAAACTTTATTCGTTTTATTTATTATATATATGAATATATA--
XVI           TTTTTACTATTTTTACATAAACTTTATTCGTTTTATTTATTATATATATGAATATATA--
XVII          TTTTTACTATTTTTACATCAT---------TTTCGTTTATTATATATATGAATATATATA
XVIII         TTTTTAC---------------------------ATTTATTATATATATGAATATATATA
Conservation  *******                            ***********************  

Bg42                                                                        420
I             CATATGAACAAAATGTACAACTAAAGAAAGTGAATCTACTTATTTAAATTCTATTTTTCT
II            CATATGAACAAAATGTACAACTAAAGAAAGTGAATCTACTTATTTAAATTCTATTTTTCT
III           CATATGAACAAAATGTACAACTAAAGAAAGTGAATCTACTTATTTAAATTCTATTTTTCT
IV            CATATGAACAAAATGTACAACTAAAGAAAGTGAATCTACTTATTTAAATTCTATTTTTCT
V             CATATGAACAAAATGTACAACTAAAGAAAGTGAATCTACTTATTTAAATTCTATTTTTCT
VI            CATATGAACAAAATGTACAACTAAAGAAAGTGAATCTACTTATTTAAATTCTATTTTTCT
VII           CATATGAACAAAATGTACAACTAAAGAAAGTGAATCTACTTATTTAAATTCTATTTTTCT
VIII          CATATGAACAAAATGTACAACTAAAGAAAGTGAATCTACTTATTTAAATTCTATTTTTCT
IX            CATATGAACAAAATGTACAACTAAAGAAAGTGAATCTACTTATTTAAATTCTATTTTTCT
X             CATATGAACAAAATGTACAACTAAAGAAAGTGAATCTACTTATTTAAATTCTATTTTTCT
XI            CATATGAACAAAATGTACAACTAAAGAAAGTGAATCTACTTATTTAAATTCTATTTTTCT
XII           CATATGAACAAAATGTACAACTAAAGAAAGTGAATCTACTTATTTAAATTCTATTTTTCT
XIII          CATATGAACAAAATGTACAACTAAAGAAAGTGAATCTACTTATTTAAATTCTATTTTTCT
XIV           CATATGAACAAAATGTACAACTAAAGAAAGTGAATCTACTTATTTAAATTCTATTTTTCT
XV            CATATGAACAAAATGTACAACTAAAGAAAGTGAATCTACTTATTTAAATTCTATTTTTCT
XVI           CATATGAACAAAATGTACAACTAAAGAAAGTGAATCTACTTATTTAAATTCTATTTTTCT
XVII          CATATGAACAAAATGTACAACTAA----------TCTACTTATTTAAATTCTATTTTTCT
XVIII         CATATGAACAAAATGTACAACTAAAGAAAGTGAATCTACTTATTTAAATTCTATTTTTCT
Conservation  ************************          **************************

Bg42                                                                        480
I             TAATCCAGGTTTAATAATATCTTGTTATGATGTTCCAATCCGCATCCGTTTT-TCCGATC
II            TAATCCAGGTTTAATAATATCTTGTTATGATGTTCCAATCCGCATCCGTTTT-TCCGATC
III           TAATCCAGGTTTAATAATATCTTGTTATGATGTTCCAATCCGCATCCGTTTT-TCCGATC
IV            TAATCCAGGTTTAATAATATCTTGTTATGATGTTCCAATCCGCATCCGTTTT-TCCGATC
V             TAATCCAGGTTTAATAATATCTTGTTATGATGTTCCAATCCGCATCCGTTTT-TCCGATC
VI            TAATCCAGGTTTAATAATATCTTGTTATGATGTTCCAATCCGCATCCGTTTT-TCCGATC
VII           TAATCCAGGTTTAATAATATCTTGTTATGATGTTCCAATCCGCATCCGTTTT-TCCGATC
VIII          TAATCCAGGTTTAATAATATCTTGTTATGATGTTCCAATCCGCATCCGTTTT-TCCGATC
IX            TAATCCAGGTTTAATAATATCTTGTTATGATGTTCCAATCCGCATCCGTTTT-TCCGATC
X             TAATCCAGGTTTAATAATATCTTGTTATGATGTTCCAATCCGCATCCGTTTT-TCCGATC
XI            TAATCCAGGTTTAATAATATCTTGTTATGATGTTCCAATCCGCATCCGTTTT-TCCGATC
XII           TAATCCAGGTTTAATAATATCTTGTTATGATGTTCCAATCCGCATCCGTTTT-TCCGATC
XIII          TAATCCAGGTTTAATAATATCTTGTTATGATGTTCCAATCCGCATCCGTTTT-TCCGATC
XIV           TAATCCAGGTTTAATAATATCTTGTTATGATGTTCCAATCCGCATCCGTTTT-TCCGATC
XV            TAATCCAGGTTTAATAATATCTTGTTATGATGTTCCAATCCGCATCCGTTTT-TCCGATC
XVI           TAATCCAGGTTTAATAATATCTTGTTATGATGTTCCAATCCGCATCCGTTTT-TCCGATC
XVII          TAATCCAGGTTTAATAATATCTTGTTATGGTGTTCCAATCCGCATCCGTTTTATCCGATC
XVIII         TAATCCAAGTTTAATAATATCTTGTTGTGGTGTTCCAATCCGCATCCGTTTTATCCGATC
Conservation  ******* ****************** ** ********************** *******
```

```
Bg42          [========================================================]  540
I             CGATCATTTTACGTCATCTGTTTTTATTATCCGTATTCGGAAAATGTTCCGGATAACGGA
II            CGATCATTTTACGTCATCTGTTTTTATTATCCGTATTCGGAAAATGTTCCGGATAACGGA
III           CGATCATTTTACGTCATCTGTTTTTATTATCCGTATTCGGAAAATGTTCCGGATAACGGA
IV            CGATCATTTTACGTCATCTGTTTTTATTATCCGTATTCGGAAAATGTTCCGGATAACGGA
V             CGATCATTTTACGTCATCTGTTTTTATTATCCGTATTCGGAAAATGTTCCGGATAACGGA
VI            CGATCATTTTACGTCATCTGTTTTTATTATCCGTATTCGGAAAATGTTCCGGATAACGGA
VII           CGATCATTTTACGTCATCTGTTTTTATTATCCGTATTCGGAAAATGTTCCGGATAACGGA
VIII          CGATCATTTTACGTCATCTGTTTTTATTATCCGTATTCGGAAAATGTTCCGGATAACGGA
IX            CGATCATTTTACGTCATCTGTTTTTATTATCCGTATTCGGAAAATGTTCCGGATAACGGA
X             CGATCATTTTACGTCATCTGTTTTTATTATCCGTATTCGGAAAATGTTCCGGATAACGGA
XI            CGATCATTTTACGTCATCTGTTTTTATTATCCGTATTCGGAAAATGTTCCGGATAACGGA
XII           CGATCATTTTACGTCATCTGTTTTTATTATCCGTATTCGGAAAATGTTCCGGATAACGGA
XIII          CGATCATTTTACGTCATCTGTTTTTATTATCCGTATTCGGAAAATGTTCCGGATAACGGA
XIV           CGATCATTTTACGTCATCTGTTTTTATTATCCGTATTCGGAAAATGTTCCGGATAACGGA
XV            CGATCATTTTACGTCATCTGTTTTTATTATCCGTATTCGGAAAATGTTCCGGATAACGGA
XVI           CGATCATTTTACGTCATCTGTTTTTATTATCCGTATTCGGAAAATGTTCCGGATAACGGA
XVII          CGATCATTTTACGTCATCTGTTTTTATTATCCGTATTCGGAAAATGTTCCGGATAACGGA
XVIII         CGATCATTTTACGTCATCTGTTTTTATTATCCGTATTCGGAAAATGTTCCGGATAACGGA
Conservation  ************************************************************

Bg42          [========================================================]  600
I             TAAACGCGTCAGTAAACTTGATGTGATAAAAATGAATTTTGTGTAAGAATGTAACGATAC
II            TAAACGCGTCAGTAAACTTGATGTGATAAAAATGAATTTTGTGTAAGAATGTAACGATAC
III           TAAACGCGTCAGTAAACTTGATGTGATAAAAATGAATTTTGTGTAAGAATGTAACGATAC
IV            TAAACGCGTCAGTAAACTTGATGTGATAAAAATGAATTTTGTGTAAGAATGTAACGATAC
V             TAAACGCGTCAGTAAACTTGATGTGATAAAAATGAATTTTGTGTAAGAATGTAACGATAC
VI            TAAACGCGTCAGTAAACTTGATGTGATAAAAATGAATTTTGTGTAAGAATGTAACGATAC
VII           TAAACGCGTCAGTAAACTTGATGTGATAAAAATGAATTTTGTGTAAGAATGTAACGATAC
VIII          TAAACGCGTCAGTAAACTTGATGTGATAAAAATGAATTTTGTGTAAGAATGTAACGATAC
IX            TAAACGCGTCAGTAAACTTGATGTGATAAAAATGAATTTTGTGTAAGAATGTAACGATAC
X             TAAACGCGTCAGTAAACTTGATGTGATAAAAATGAATTTTGTGTAAGAATGTAACGATAC
XI            TAAACGCGTCAGTAAACTTGATGTGATAAAAATGAATTTTGTGTAAGAATGTAACGATAC
XII           TAAACGCGTCAGTAAACTTGATGTGATAAAAATGAATTTTGTGTAAGAATGTAACGATAC
XIII          TAAACGCGTCAGTAAACTTGATGTGATAAAAATGAATTTTGTGTAAGAATGTAACGATAC
XIV           TAAACGCGTCAGTAAACTTGATGTGATAAAAATGAATTTTGTGTAAGAATGTAACGATAC
XV            TAAACGCGTCAGTAAACTTGATGTGATAAAAATGAATTTTGTGTAAGAATGTAACGATAC
XVI           TAAACGCGTCAGTAAACTTGATGTGATAAAAATGAATTTTGTGTAAGAATGTAACGATAC
XVII          TAAACGCGTCAGTAAACTTGATGTGATAAAAATGAATTTTGTGTAAGAATGTAACGATAC
XVIII         TAAACGCGTCAGTAAACTTGATGTGATAAAAATGAATTTTGTGTAAGAATGTAACGATAC
Conservation  ************************************************************

Bg62          [========================================================]  660
I             ACATAAATTACGAAAGTAAAAGTAATAGAAAAAAATAAAAAAAATAAAATAAAATAAAA-
II            ACATAAATTACGAAAGTAAAAGTAATAGAAAAAAATAAAAAAAATAAAATAAAATAAAA-
III           ACATAAATTACGAAAGTAAAAGTAATAGAAAAAAATAAAAAAAATAAAATAAAATAAAA-
IV            ACATAAATTACGAAAGTAAAAGTAATAGAAAAAAATAAAAAAAATAAAATAAAATAAAA-
V             ACATAAATTACGAAAGTAAAAGTAATAGAAAAAAATAAAAAAAATAAAATAAAATAAAA-
VI            ACATAAATTACGAAAGTAAAAGTAATAGAAAAAAATAAAAAAAATAAAATAAAATAAAA-
VII           ACATAAATTACGAAAGTAAAAGTAATAGAAAAAAATAAAAAAAATAAAATAAAATAAAA-
VIII          ACATAAATTACGAAAGTAAAAGTAATAGAAAAAAATAAAAAAAATAAAATAAAATAAAA-
IX            ACATAAATTACGAAAGTAAAAGTAATAGAAAAAAATAAAAAAAATAAAATAAAATAAAA-
X             ACATAAATTACGAAAGTAAAAGTAATAGAAAAAAATAAAAAAAATAAAATAAAATAAAA-
XI            ACATAAATTACGAAAGTAAAAGTAATAGAAAAAAATAAAAAAAATAAAATAAAATAAAA-
X             ACATAAATTACGAAAGTAAAAGTAATAGAAAAAAATAAAAAAAATAAAATAAAATAAAA-
XII           ACATAAATTACGAAAGTAAAAGTAATAGAAAAAAATAAAAAAAATAAAATAAAATAAAA-
XIII          ACATAAATTACGAAAGTAAAAGTAATAGAAAAAAATAAAAAAAATAAAATAAAATAAAA-
XIV           ACATAAATTACGAAAGTAAAAGTAATAGAAAAAAATAAAAAAAATAAAATAAAATAAAA-
XV            ACATAAATTACGAAAGTAAAAGTAATAGAAAAAAATAAAAAAAATAAAATAAAATAAAAT
XVI           ACATAAATTACGAAAGTAAAAGTAATAGAAAAAAATAAAAAAAATAAAATAAAATAAAAT
XVII          ACATAAATTACGAAAGTAAAAGTAATAGAAAAAAATAAAAAAAATAAAATAAAATAAAA-
XVIII         ACATAAATTACGAAAGTAAAAGTAATAGAAAAAAATAAAAAAAATAAAATAAAATAAAA-
Conservation  *********************************************************** 

Bg62          [========================================================]  720
I             ---------TAAAATAAAATAAAATAAAATAAATATAATTTAAGAATTTAAAGAACATTT
II            ---------TAAAATAAAATAAAATAAAATAAATATAATTTAAGAATTTAAAGAACATTT
III           ---------TAAAATAAAATAAAATAAAATAAATATAATTTAAGAATTTAAAGAACATTT
IV            ---------TAAAATAAAATAAAATAAAATAAATATAATTTAAGAATTTAAAGAACATTT
V             ---------TAAAATAAAATAAAATAAAATAAATATAATTTAAGAATTTAAAGAACATTT
VI            ---------TAAAATAAAATAAAATAAAATAAATATAATTTAAGAATTTAAAGAACATTT
VII           ---------TAAAATAAAATAAAATAAAATAAATATAATTTAAGAATTTAAAGAACATTT
VIII          ---------TAAAATAAAATAAAATAAAATAAATATAATTTAAGAATTTAAAGAACATTT
IX            ---------TAAAATAAAATAAAATAAAATAAATATAATTTAAGAATTTAAAGAACATTT
X             ---------TAAAATAAAATAAAATAAAATAAATATAATTTAAGAATTTAAAGAACATTT
XI            ---------TAAAATAAAATAAAATAAAATAAATATAATTTAAGAATTTAAAGAACATTT
XII           ---------TAAAATAAAATAAA----------TATAATTTAAGAATTTAAAGAACATTT
XIII          ---------TAAAATAAAATAAAATAAAATAAATATAATTTAAGAATTTAAAGAACATTT
XIV           ---------TAAAATAAAATAAAATAAAATAAATATAATTTAAGAATTTAAAGAACATTT
XV            AAAATAAAATAAAATAAAATAAAATAAAATAAATATAATTTAAGAATTTAAAGAACATTT
XVI           AAAATAAAATAAAATAAAATAAAATAAAATAAATATAATTTAAGAATTTAAAGAACATTT
XVII          -------------------TAAAATAAAATAAATATAATTTAAGAATTTAAAGAACATTT
XVIII         --------------TAAAATAAAATAAAATAAATATAATTTAAGAATTTAAAGAACATTT
Conservation                     ****          ***************************
```

```
Bg62                                                                   780
I             TTATAGGTAAAATTCAACTTTCTTTCTTTTTTTTGTTTTTAATAAATATAAATAAAAAAG
II            TTATAGGTAAAATTCAACTTTCTTTCTTTTTTTTGTTTTTAATAAATATAAATAAAAAAG
III           TTATAGGTAAAATTCAACTTTCTTTCTTTTTTTTGTTTTTAATAAATATAAATAAAAAAG
IV            TTATAGGTAAAATTCAACTTTCTTTCTTTTTTTTGTTTTTAATAAATATAAATAAAAAAG
V             TTATAGGTAAAATTCAACTTTCTTTCTTTTTTTTGTTTTTAATAAATATAAATAAAAAAG
VI            TTATAGGTAAAATTCAACTTTCTTTCTTTTTTTTGTTTTTAATAAATATAAATAAAAAAG
VII           TTATAGGTAAAATTCAACTTTCTTTCTTTTTTTTGTTTTTAATAAATATAAATAAAAAAG
VIII          TTATAGGTAAAATTCAACTTTCTTTCTTTTTTTTGTTTTTAATAAATATAAATAAAAAAG
IX            TTATAGGTAAAATTCAACTTTCTTTCTTTTTTTTGTTTTTAATAAATATAAATAAAAAAG
X             TTATAGGTAAAATTCAACTTTCTTTCTTTTTTTTGTTTTTAATAAATATAAATAAAAAAG
XI            TTATAGGTAAAATTCAACTTTCTTTCTTTTTTTTGTTTTTAATAAATATAAATAAAAAAG
XII           TTATAGGTAAAATTCAACTTTCTTTCTTTTTTTTGTTTTTAATAAATATAAATAAAAAAG
XIII          TTATAGGTAAAATTCAACTTTCTTTCTTTTTTTTGTTTTTAATAAATATAAATAAAAAAG
XIV           TTATAGGTAAAATTCAACTTTCTTTCTTTTTTTTGTTTTTAATAAATATAAATAAAAAAG
XV            TTATAGGTAAAATTCAACTTTCTTTCTTTTTT--------AATAAATATAAATAAAAAAG
XVI           TTATAGGTAAAATTCAACTTTCTTTCTTTTTT--------AATAAATATAAATAAAAAAG
XVII          TTATAGGTAAAATTCAACTTTCTTTCTTTTTTTTGTTTTTAATAAATATAAATAAAAAAG
XVIII         TTATAGGTAAAATTCAACTTTCTTTCTTTTTTTTGTTTTTAATAAATATAAATAAAAAAG
Conservation  ********************************        ********************
```

```
Bg62                                                                   840
I             GGGAATTATAAATATTACTAATATAAAATACTTTACCTATATGAAATACTGGTTTTGCTA
II            GGGAATTATAAATATTACTAATATAAAATACTTTACCTATATGAAATACTGGTTTTGCTA
III           GGGAATTATAAATATTACTAATATAAAATACTTTACCTATATGAAATACTGGTTTTGCTA
IV            GGGAATTATAAATATTACTAATATAAAATACTTTACCTATATGAAATACTGGTTTTGCTA
V             GGGAATTATAAATATTACTAATATAAAATACTTTACCTATATGAAATACTGGTTTTGCTA
VI            GGGAATTATAAATATTACTAATATAAAATACTTTACCTATATGAAATACTGGTTTTGCTA
VII           GGGAATTATAAATATTACTAATATAAAATACTTTACCTATATGAAATACTGGTTTTGCTA
VIII          GGGAATTATAAATATTACTAATATAAAATACTTTACCTATATGAAATACTGGTTTTGCTA
IX            GGGAATTATAAATATTACTAATATAAAATACTTTACCTATATGAAATACTGGTTTTGCTA
X             GGGAATTATAAATATTACTAATATAAAATACTTTACCTATATGAAATACTGGTTTTGCTA
XI            GGGAATTATAAATATTACTAATATAAAATACTTTACCTATATGAAATACTGGTTTTGCTA
XII           GGGAATTATAAATATTACTAATATAAAATACTTTACCTATATGAAATACTGGTTTTGCTA
XIII          GGGAATTATAAATATTACTAATATAAAATACTTTACCTATATGAAATACTGGTTTTGCTA
XIV           GGGAATTATAAATATTACTAATATAAAATACTTTACCTATATGAAATACTGGTTTTGCTA
XV            GGGAATTATAAATATTACTAATATAAAATACTTTACCTATATGAAATACTGGTTTTGCTA
XVI           GGGAATTATAAATATTACTAATATAAAATACTTTACCTATATGAAATACTGGTTTTGCTA
XVII          GGGAATTATAAATATTACTAATATAAAATACTTTACCTATATGAAATACTGGTTTTGCTA
XVIII         GGGAATTATAAATATTACTAATATAAAATACTTTACCTATATGAAATACTGGTTTTGCTA
Conservation  ************************************************************
```

```
Bg62                                                                   900
I             GTTCTATGTGTCCAAAGTGGCTTATTACTTGGAATGGACACGCAGATCGTAAAGTAAATT
II            GTTCTATGTGTCCAAAGTGGCTTATTACTTGGAATGGACACGCAGATCGTAAAGTAAATT
III           GTTCTATGTGTCCAAAGTGGCTTATTACTTGGAATGGACACGCAGATCGTAAAGTAAATT
IV            GTTCTATGTGTCCAAAGTGGCTTATTACTTGGAATGGACACGCAGATCGTAAAGTAAATT
V             GTTCTATGTGTCCAAAGTGGCTTATTACTTGGAATGGACACGCAGATCGTAAAGTAAATT
VI            GTTCTATGTGTCCAAAGTGGCTTATTACTTGGAATGGACACGCAGATCGTAAAGTAAATT
VII           GTTCTATGTGTCCAAAGTGGCTTATTACTTGGAATGGACACGCAGATCGTAAAGTAAATT
VIII          GTTCTATGTGTCCAAAGTGGCTTATTACTTGGAATGGACACGCAGATCGTAAAGTAAATT
IX            GTTCTATGTGTCCAAAGTGGCTTATTACTTGGAATGGACACGCAGATCGTAAAGTAAATT
X             GTTCTATGTGTCCAAAGTGGCTTATTACTTGGAATGGACACGCAGATCGTAAAGTAAATT
XI            GTTCTATGTGTCCAAAGTGGCTTATTACTTGGAATGGACACGCAGATCGTAAAGTAAATT
XII           GTTCTATGTGTCCAAAGTGGCTTATTACTTGGAATGGACACGCAGATCGTAAAGTAAATT
XIII          GTTCTATGTGTCCAAAGTGGCTTATTACTTGGAATGGACACGCAGATCGTAAAGTAAATT
XIV           GTTCTATGTGTCCAAAGTGGCTTATTACTTGGAATGGACACGCAGATCGTAAAGTAAATT
XV            GTTCTATGTGTCCAAAGTGGCTTATTACTTGGAATGGACACGCAGATCGTAAAGTAAATT
XVI           GTTCTATGTGTCCAAAGTGGCTTATTACTTGGAATGGACACGCAGATCGTAAAGTAAATT
XVII          GTTCTATGTGTCCAAAGTGGCTTATTACTTGGAATGGACACGCAGATCGTAAAGTAAATT
XVIII         GTTCTATGTGTCCAAAGTGGCTTATTACTTGGAATGGACACGCAGATCGTAAAGTAAATT
Conservation  ************************************************************
```

```
Bg196                                                                  960
I             TGTGATTTTATTTTATT----------AGAGATTTTATGTAATAAAATAAGTATATTGAA
II            TGTGATTTTATTTTATTTTATTTTATTGAAGATTTCATGTAATAAAATAAGTATATTGAA
III           TGTGATTTTATTTTATTTTATTTTATTGAAGATTTCATGTAATAAAATAAGTATATTGAA
IV            TGTGATTTTATTTTATT----------AGAGATTTTATGTAATAAAATAAGTATATTGAA
V             TGTGATTTTATTTTATT----------AGAGATTTTATGTAATAAAATAAGTATATTGAA
VI            TGTGATTTTATTTTATT----------AGAGATTTTATGTAATAAAATAAGTATATTGAA
VII           TGTGATTTTATTTTATT----------AGAGATTTTATGTAATAAAATAAGTATATTGAA
VIII          TGTGATTTTATTTTATT----------AGAGATTTTATGTAATAAAATAAGTATATTGAA
IX            TGTGATTTTATTTTATT----------AGAGATTTTATGTAATAAAATAAGTATATTGAA
X             TGTGATTTTATTTTATT----------AGAGATTTTATGTAATAAAATAAGTATATTGAA
XI            TGTGATTTTATTTTATT----------AGAGATTTTATGTAATAAAATAAGTATATTGAA
XII           TGTGATTTTATTTTATTTTATTTTATTGAAGATTTCATGTAATAAAATAAGTATATTGAA
XIII          TGTGATTTTATTTTATTTTATTTTATTGAAGATTTCATGTAATAAAATAAGTATATTGAA
XIV           TGTGATTTTATTTTATTTTATTTTATTGAAGATTTCATGTAATAAAATAAGTATATTGAA
XV            TGTGATTTTATTTTATT----------AGAGATTTTATGTAATAAAATAAGTATATTGAA
XVI           TGTGATTTTATTTTATT----------AGAGATTTTATGTAATAAAATAAGTATATTGAA
XVII          TGTGATTTTATTTTATT----------AGAGATTTTATGTAATAAAAT--------TGAA
XVIII         TGTGATTTTATTTTATT----------AGAGATTTTATGTAATAAAATAAGTATATTGAA
Conservation  *****************            ****** ************        ****
```

```
Bg196                                                                  1020
I             AAAAAATTTTATTTACAGTATATATAATATTTAATAATAATTTTTTAAACAAAAATGATA
II            AAAAA-TTTTATTTACAGTATATATAATATTTAATAATAAGTTTTTATATAAAAATGGTA
III           AAAAA-TTTTATTTACAGTATATATAATATTTAATAATAAGTTTTTATATAAAAATGGTA
IV            AAAAAATTTTATTTACAGTATATATAATATTTAATAATAATTTTTTAAACAAAAATGATA
V             AAAAAATTTTATTTACAGTATATATAATATTTAATAATAATTTTTTAAACAAAAATGATA
VI            AAAAAATTTTATTTACAGTATATATAATATTTAATAATAATTTTTTAAACAAAAATGATA
VII           AAAAAATTTTATTTACAGTATATATAATATTTAATAATAATTTTTTAAACAAAAATGATA
VIII          AAAAAATTTTATTTACAGTATATATAATATTTAATAATAATTTTTTAAACAAAAATGATA
IX            AAAAAATTTTATTTACAGTATATATAATATTTAATAATAATTTTTTAAACAAAAATGATA
X             AAAAAATTTTATTTACAGTATATATAATATTTAATAATAATTTTTTAAACAAAAATGATA
XI            AAAAAATTTTATTTACAGTATATATAATATTTAATAATAATTTTTTAAACAAAAATGATA
XII           AAAAA-TTTTATTTACAGTATATATAATATTTAATAATAAGTTTTTATATAAAAATGGTA
XIII          AAAAA-TTTTATTTACAGTATATATAATATTTAATAATAAGTTTTTATATAAAAATGGTA
XIV           AAAAA-TTTTATTTACAGTATATATAATATTTAATAATAAGTTTTTATATAAAAATGGTA
XV            AAAAAATTTTATTTACAGTATATATAATATTTAATAATAATTTTTTAAACAAAAATGATA
XVI           AAAAAATTTTATTTACAGTATATATAATATTTAATAATAATTTTTTAAACAAAAATGATA
XVII          AAAAAATTTTATTTACAGTATATATAATATTTAATAATAATTTTTTAAATAAAAATGATA
XVIII         AAAAAATTTT---TACAGTATATATAATATTTAA-------------------AATGATA
Conservation  ***** ****   *********************                   **** **

Bg196                                                                  1080
I             AATAATAGAAATAAAGCTTGCAAGTAATAGCGAATTATAAAAATAGTTATTATGTATTAT
II            AATAATAGAT-TAAAACTTGCAAGTAATAGCGAATTATAAAAATAATTATTATGTATTAT
III           AATAATAGAT-TAAAACTTGCAAGTAATAGCGAATTATAAAAATAATTATTATGTATTAT
IV            AATAATAGAAATAAAGCTTGCAAGTAATAGCGAATTATAAAAATAGTTATTATGTATTAT
V             AATAATAGAAATAAAGCTTGCAAGTAATAGCGAATTATAAAAATAGTTATTATGTATTAT
VI            AATAATAGAAATAAAGCTTGCAAGTAATAGCGAATTATAAAAATAGTTATTATGTATTAT
VII           AATAATAGAAATAAAGCTTGCAAGTAATAGCGAATTATAAAAATAGTTATTATGTATTAT
VIII          AATAATAGAAATAAAGCTTGCAAGTAATAGCGAATTATAAAAATAGTTATTATGTATTAT
IX            AATAATAGAAATAAAGCTTGCAAGTAATAGCGAATTATAAAAATAGTTATTATGTATTAT
X             AATAATAGAAATAAAGCTTGCAAGTAATAGCGAATTATAAAAATAGTTATTATGTATTAT
XI            AATAATAGAAATAAAGCTTGCAAGTAATAGCGAATTATAAAAATAGTTATTATGTATTAT
XII           AATAATAGAT-TAAAACTTGCAAGTAATAGCGAATTATAAAAATAATTATTATGTATTAT
XIII          AATAATAGAT-TAAAACTTGCAAGTAATAGCGAATTATAAAAATAATTATTATGTATTAT
XIV           AATAATAGAT-TAAAACTTGCAAGTAATAGCGAATTATAAAAATAATTATTATGTATTAT
XV            AATAATAGAAATAAAGCTTGCAAGTA---GCGAATTATAAAAATAGTTATTATGTATTAT
XVI           AATAATAGAAATAAAGCTTGCAAGTA---GCGAATTATAAAAATAGTTATTATGTATTAT
XVII          AATAATAGAAATAAAGCTTGCAAGTAATAGCGAATTATAAAAATAGTTATTATGTATTAT
XVIII         AATAATAGAAATAAAGCTTGCAAGTAATAGCGAATTATAAAAATAGTTATTATGTATTAT
Conservation  *********  **** **********   **************** **************

Bg196                                                                  1140
I             T-GATTAAGATGTAAAATTTAATAATATCTCTGATAGCAAGGGGAGCTTATTGAAGAGTC
II            TTGATTAAGATGTAAAATTTAATAATATCTCTGATAGCAAGGGGAGCTTATTGAAGAGTC
III           TTGATTAAGATGTAAAATTTAATAATATCTCTGATAGCAAGGGGAGCTTATTGAAGAGTC
IV            T-GATTAAGATGTAAAATTTAATAATATCTCTGATAGCAAGGGGAGCTTATTGAAGAGTC
V             T-GATTAAGATGTAAAATTTAATAATATCTCTGATAGCAAGGGGAGCTTATTGAAGAGTC
VI            T-GATTAAGATGTAAAATTTAATAATATCTCTGATAGCAAGGGGAGCTTATTGAAGAGTC
VII           T-GATTAAGATGTAAAATTTAATAATATCTCTGATAGCAAGGGGAGCTTATTGAAGAGTC
VIII          T-GATTAAGATGTAAAATTTAATAATATCTCTGATAGCAAGGGGAGCTTATTGAAGAGTC
IX            T-GATTAAGATGTAAAATTTAATAATATCTCTGATAGCAAGGGGAGCTTATTGAAGAGTC
X             T-GATTAAGATGTAAAATTTAATAATATCTCTGATAGCAAGGGGAGCTTATTGAAGAGTC
XI            T-GATTAAGATGTAAAATTTAATAATATCTCTGATAGCAAGGGGAGCTTATTGAAGAGTC
XII           TTGATTAAGATGTAAAATTTAATAATATCTCTGATAGCAAGGGGAGCTTATTGAAGAGTC
XIII          TTGATTAAGATGTAAAATTTAATAATATCTCTGATAGCAAGGGGAGCTTATTGAAGAGTC
XIV           TTGATTAAGATGTAAAATTTAATAATATCTCTGATAGCAAGGGGAGCTTATTGAAGAGTC
XV            T-GATTAAGATGTAAAATTTAATAATATCTCTGATAGCAAGGGGAGCTTATTGAAGAGTC
XVI           T-GATTAAGATGTAAAATTTAATAATATCTCTGATAGCAAGGGGAGCTTATTGAAGAGTC
XVII          T-GATTAAGGTGTAAAATTTAATAATATCTCTGATAGCAAGGGGAGCTTATTGAAGAGTC
XVIII         T-GATTAAGGTGTAAAATTTAATAATATCTCTGATAGCAAGGGGAGCTTATTGAAGAGTC
Conservation  * ******* **************************************************

Bg196                                                                  1200
I             AAGAGAATAGGTACAGATTCGTATTGGATATTAGCCCATTTACATCACTTTTGCTTATAT
II            AAGAGAATAGGTACAGATTCGTATTGGATATTAGCCCATTTACATCACTTTTGCTTATAT
III           AAGAGAATAGGTACAGATTCGTATTGGATATTAGCCCATTTACATCACTTTTGCTTATAT
IV            AAGAGAATAGGTACAGATTCGTATTGGATATTAGCCCATTTACATCACTTTTGCTTATAT
V             AAGAGAATAGGTACAGATTCGTATTGGATATTAGCCCATTTACATCACTTTTGCTTATAT
VI            AAGAGAATAGGTACAGATTCGTATTGGATATTAGCCCATTTACATCACTTTTGCTTATAT
VII           AAGAGAATAGGTACAGATTCGTATTGGATATTAGCCCATTTACATCACTTTTGCTTATAT
VIII          AAGAGAATAGGTACAGATTCGTATTGGATATTAGCCCATTTACATCACTTTTGCTTATAT
IX            AAGAGAATAGGTACAGATTCGTATTGGATATTAGCCCATTTACATCACTTTTGCTTATAT
X             AAGAGAATAGGTACAGATTCGTATTGGATATTAGCCCATTTACATCACTTTTGCTTATAT
XI            AAGAGAATAGGTACAGATTCGTATTGGATATTAGCCCATTTACATCACTTTTGCTTATAT
XII           AAGAGAATAGGTACAGATTCGTATTGGATATTAGCCCATTTACATCACTTTTGCTTATAT
XIII          AAGAGAATAGGTACAGATTCGTATTGGATATTAGCCCATTTACATCACTTTTGCTTATAT
XIV           AAGAGAATAGGTACAGATTCGTATTGGATATTAGCCCATTTACATCACTTTTGCTTATAT
XV            AAGAGAATAGGTACAGATTCGTATTGGATATTAGCCCATTTACATCACTTTTGCTTATAT
XVI           AAGAGAATAGGTACAGATTCGTATTGGATATTAGCCCATTTACATCACTTTTGCTTATAT
XVII          AAGAGAATAGGTACAGATTCGTATTGGATATTAGCCCATTTACATCACTTTTGCTTATAT
XVIII         AAGAGAATAGGTACAGATTCGTATTGGATATTAGCCCATTTACATCACTTTTGCTTATAT
Conservation  ************************************************************
```

```
Bg235                                                                     1260
I             GTTCACTATATATCGTCATACATCATCTTATAATTTTATCATAAAAGATACAGCATAATG
II            GTTCACTATATATCGTCATACATCATCTTATAATTTTATCATAAAAGATACAGCATAATG
III           GTTCACTATATATCGTCATACATCATCTTATAATTTTATCATAAAAGATACAGCATAATG
IV            GTTCACTATATATCGTCATACATCATCTTATAATTTTATCATAAAAGATACAGCATAATG
V             GTTCACTATATATCGTCATACATCATCTTATAATTTTATCATAAAAGATACAGCATAATG
VI            GTTCACTATATATCGTCATACATCATCTTATAATTTTATCATAAAAGATACAGCATAATG
VII           GTTCACTATATATCGTCATACATCATCTTATAATTTTATCATAAAAGATACAGCATAATG
VIII          GTTCACTATATATCGTCATACATCATCTTATAATTTTATCATAAAAGATACAGCATAATG
IX            GTTCACTATATATCGTCATACATCATCTTATAATTTTATCATAAAAGATACAGCATAATG
X             GTTCACTATATATCGTCATACATCATCTTATAATTTTATCATAAAAGATACAGCATAATG
XI            GTTCACTATATATCGTCATACATCATCTTATAATTTTATCATAAAAGATACAGCATAATG
XII           GTTCACTATATATCGTCATACATCATCTTATAATTTTATCATAAAAGATACAGCATAATG
XIII          GTTCACTATATATCGTCATACATCATCTTATAATTTTATCATAAAAGATACAGCATAATG
XIV           GTTCACTATATATCGTCATACATCATCTTATAATTTTATCATAAAAGATACAGCATAATG
XV            GTTCACTATATATCGTCATACATCATCTTATAATTTTATCATAAAAGATACAGCATAATG
XVI           GTTCACTATATATCGTCATACATCATCTTATAATTTTATCATAAAAGATACAGCATAATG
XVII          GTTCACTATATATCGTCATACATCATCTTATAATTTTATCATAAAAGATACAGCATAATG
XVIII         GTTCACTATATATCGTCATACATCATCTTATAATTTTATCATAAAAGATACAGCATAATG
Conservation  ************************************************************

Bg235                                                                     1320
I             GAAGTTCTATTTTGACCTATTATTATTACACAAACTATTTA-----TACAGGGTATACAA
II            GAAGTTCTATTTTGACCTATTATTATTACACAAACTATTTA-----TACAGGGTATACAA
III           GAAGTTCTATTTTGACCTATTATTATTACACAAACTATTTA-----TACAGGGTATACAA
IV            GAAGTTCTATTTTGACCTATTATTATTACACAAACTATTTA-----TACAGGGTATACAA
V             GAAGTTCTATTTTGACCTATTATTATTACACAAACTATTTA-----TACAGGGTATACAA
VI            GAAGTTCTATTTTGACCTATTATTATTACACAAACTATTTA-----TACAGGGTATACAA
VII           GAAGTTCTATTTTGACCTATTATTATTACACAAACTATTTA-----TACAGGGTATACAA
VIII          GAAGTTCTATTTTGACCTATTATTATTACACAAACTATTTA-----TACAGGGTATACAA
IX            GAAGTTCTATTTTGACCTATTATTATTACACAAACTATTTA-----TACAGGGTATACAA
X             GAAGTTCTATTTTGACCTATTATTATTACACAAACTATTTA-----TACAGGGTATACAA
XI            GAAGTTCTATTTTGACCTATTATTATTACACAAACTATTTA-----TACAGGGTATACAA
XII           GAAGTTCTATTTTGACCTATTATTATTACACAAACTATTTA-----TACAGGGTATACAA
XIII          GAAGTTCTATTTTGACCTATTATTATTACACAAACTATTTA-----TACAGGGTATACAA
XIV           GAAGTTCTATTTTGACCTATTATTATTACACAAACTATTTA-----TACAGGGTATACAA
XV            GAAGTTCTATTTTGACCTATTATTATTACACAAACTATTTA-----TACAGGGTATACAA
XVI           GAAGTTCTATTTTGACCTATTATTATTACACAAACTATTTA-----TACAGGGTATACAA
XVII          GAAGTTCTATTTTGATCTATTATTATTACACAAACTATTTACAGTATACAGTGTATACAA
XVIII         GAAGTTCTATTTTGATCTATTATTATTACACAAAGT----------------GTATACAA
Conservation  *************** ****************** *                ********

Bg235                                                                     1380
I             CAGTATACATATATGATTTACGATTAACCACTTGAAAAACTTAACCAATTACTCTGATAA
II            CAGTATACATATATGATTTACGATTAACCACTTGAAAAACTTAACCAATTACTCTGATAA
III           CAGTATACATATATGATTTACGATTAACCACTTGAAAAACTTAACCAATTACTCTGATAA
IV            CAGTATACATATATGATTTACGATTAACCACTTGAAAAACTTAACCAATTACTCTGATAA
V             CAGTATACATATATGATTTACGATTAACCACTTGAAAAACTTAACCAATTACTCTGATAA
VI            CAGTATACATATATGATTTACGATTAACCACTTGAAAAACTTAACCAATTACTCTGATAA
VII           CAGTATACATATATGATTTACGATTAACCACTTGAAAAACTTAACCAATTACTCTGATAA
VIII          CAGTATACATATATGATTTACGATTAACCACTTGAAAAACTTAACCAATTACTCTGATAA
IX            CAGTATACATATATGATTTACGATTAACCACTTGAAAAACTTAACCAATTACTCTGATAA
X             CAGTATACATATATGATTTACGATTAACCACTTGAAAAACTTAACCAATTACTCTGATAA
XI            CAGTATACATATATGATTTACGATTAACCACTTGAAAAACTTAACCAATTACTCTGATAA
XII           CAGTATACATATATGATTTACGATTAACCACTTGAAAAACTTAACCAATTACTCTGATAA
XIII          CAGTATACATATATGATTTACGATTAACCACTTGAAAAACTTAACCAATTACTCTGATAA
XIV           CAGTATACATATATGATTTACGATTAACCACTTGAAAAACTTAACCAATTACTCTGATAA
XV            CAGTATACATATATGATTTACGATTAACCACTTGAAAAACTTAACCAATTACTCTGATAA
XVI           CAGTATACATATATGATTTACGATTAACCACTTGAAAAACTTAACCAATTACTCTGATAA
XVII          CAGTATAC--------------------CACTTGAAAAACTTAACCAATTACTCTGATAA
XVIII         CAGTATACATATATGATTTATGATCAACCACTTGAAAAACTTAACCAATTACTCTGATAA
Conservation  ********                    ********************************

Bg235                                                                     1440
I             AATATGAA-TATAAAGTATAACTACCAGCTATCTATCCTGTTGATATAAACAACCAATTA
II            AATATGAA-TATAAAGTATAACTACCAGCTATCTATCCTGTTGATATAAACAACCAATTA
III           AATATGAA-TATAAAGTATAACTACCAGCTATCTATCCTGTTGATATAAACAACCAATTA
IV            AATATGAA-TATAAAGTATAACTACCAGCTATCTATCCTGTTGATATAAACAACCAATTA
V             AATATGAA-TATAAAGTATAACTACCAGCTATCTATCCTGTTGATATAAACAACCAATTA
VI            AATATGAA-TATAAAGTATAACTACCAGCTATCTATCCTGTTGATATAAACAACCAATTA
VII           AATATGAA-TATAAAGTATAACTACCAGCTATCTATCCTGTTGATATAAACAACCAATTA
VIII          AATATGAA-TATAAAGTATAACTACCAGCTATCTATCCTGTTGATATAAACAACCAATTA
IX            AATATGAA-TATAAAGTATAACTACCAGCTATCTATCCTGTTGATATAAACAACCAATTA
X             AATATGAA-TATAAAGTATAACTACCAGCTATCTATCCTGTTGATATAAACAACCAATTA
XI            AATATGAA-TATAAAGTATAACTACCAGCTATCTATCCTGTTGATATAAACAACCAATTA
XII           AATATGAA-TATAAAGTATAACTACCAGCTATCTATCCTGTTGATATAAACAACCAATTA
XIII          AATATGAA-TATAAAGTATAACTACCAGCTATCTATCCTGTTGATATAAACAACCAATTA
XIV           AATATGAA-TATAAAGTATAACTACCAGCTATCTATCCTGTTGATATAAACAACCAATTA
XV            AATATGAA-TATAAAGTATAACTACCAGCTATCTATCCTGTTGATATAAACAACCAATTA
XVI           AATATGAA-TATAAAGTATAACTACCAGCTATCTATCCTGTTGATATAAACAACCAATTA
XVII          AATATGAAATATAAAGTATAACTACCAGCTATCCATCCTGTTGATAACAAC--CCAATTA
XVIII         AATATGAAATATAAAGTATAACTACCAGCTATCCATCCTGTTGATAACAAC--CCAATTA
Conservation  ******** ************************ ************  ***  *******
```

```
Bg235                                                                          1500
I             AACATGATTTGTTGTAAAATGCTTGTTTACATATGTAGGATTACTTATCTGAAGCCACAT
II            AACATGATTTGTTGTAAAATGCTTGTTTACATATGTAGGATTACTTATCTGAAGCCACAT
III           AACATGATTTGTTGTAAAATGCTTGTTTACATATGTAGGATTACTTATCTGAAGCCACAT
IV            AACATGATTTGTTGTAAAATGCTTGTTTACATATGTAGGATTACTTATCTGAAGCCACAT
V             AACATGATTTGTTGTAAAATGCTTGTTTACATATGTAGGATTACTTATCTGAAGCCACAT
VI            AACATGATTTGTTGTAAAATGCTTGTTTACATATGTAGGATTACTTATCTGAAGCCACAT
VII           AACATGATTTGTTGTAAAATGCTTGTTTACATATGTAGGATTACTTATCTGAAGCCACAT
VIII          AACATGATTTGTTGTAAAATGCTTGTTTACATATGTAGGATTACTTATCTGAAGCCACAT
IX            AACATGATTTGTTGTAAAATGCTTGTTTACATATGTAGGATTACTTATCTGAAGCCACAT
X             AACATGATTTGTTGTAAAATGCTTGTTTACATATGTAGGATTACTTATCTGAAGCCACAT
XI            AACATGATTTGTTGTAAAATGCTTGTTTACATATGTAGGATTACTTATCTGAAGCCACAT
XII           AACATGATTTGTTGTAAAATGCTTGTTTACATATGTAGGATTACTTATCTGAAGCCACAT
XIII          AACATGATTTGTTGTAAAATGCTTGTTTACATATGTAGGATTACTTATCTGAAGCCACAT
XIV           AACATGATTTGTTGTAAAATGCTTGTTTACATATGTAGGATTACTTATCTGAAGCCACAT
XV            AACATGATTTGTTGTAAAATGCTTGTTTACATATGTAGGATTACTTATCTGAAGCCACAT
XVI           AACATGATTTGTTGTAAAATGCTTGTTTACATATGTAGGATTACTTATCTGAAGCCACAT
XVII          AACATGATTTGTTGTAAAATGCTTATCTACATATGTAGGATTACTTATCTGAAGCCACAT
XVIII         AACATGATTTGTTGTAAAATGCTTGTCTACATA----------CTTATCTGAAGCCACAT
Conservation  ************************ * ******          *****************

Bg235                                                                          1560
I             GATCTCACAGGCAATTCGGGTAGATACAATTTTGGTCGATTATTTAAAAATCTAAGTAAC
II            GATCTCACAGGCAATTCGGGTAGATACAATTTTGGTCGATTATTTAAAAATCTAAGTAAC
III           GATCTCACAGGCAATTCGGGTAGATACAATTTTGGTCGATTATTTAAAAATCTAAGTAAC
IV            GATCTCACAGGCAATTCGGGTAGATACAATTTTGGTCGATTATTTAAAAATCTAAGTAAC
V             GATCTCACAGGCAATTCGGGTAGATACAATTTTGGTCGATTATTTAAAAATCTAAGTAAC
VI            GATCTCACAGGCAATTCGGGTAGATACAATTTTGGTCGATTATTTAAAAATCTAAGTAAC
VII           GATCTCACAGGCAATTCGGGTAGATACAATTTTGGTCGATTATTTAAAAATCTAAGTAAC
VIII          GATCTCACAGGCAATTCGGGTAGATACAATTTTGGTCGATTATTTAAAAATCTAAGTAAC
IX            GATCTCACAGGCAATTCGGGTAGATACAATTTTGGTCGATTATTTAAAAATCTAAGTAAC
X             GATCTCACAGGCAATTCGGGTAGATACAATTTTGGTCGATTATTTAAAAATCTAAGTAAC
XI            GATCTCACAGGCAATTCGGGTAGATACAATTTTGGTCGATTATTTAAAAATCTAAGTAAC
XII           GATCTCACAGGCAATTCGGGTAGATACAATTTTGGTCGATTATTTAAAAATCTAAGTAAC
XIII          GATCTCACAGGCAATTCGGGTAGATACAATTTTGGTCGATTATTTAAAAATCTAAGTAAC
XIV           GATCTCACAGGCAATTCGGGTAGATACAATTTTGGTCGATTATTTAAAAATCTAAGTAAC
XV            GATCTCACAGGCAATTCGGGTAGATACAATTTTGGTCGATTATTTAAAAATCTAAGTAAC
XVI           GATCTCACAGGCAATTCGGGTAGATACAATTTTGGTCGATTATTTAAAAATCTAAGTAAC
XVII          GATCTCACAGGCAATTCGGGTAGATACAATTTTGGTCGATTATTTAAAAATCTAAGTAAC
XVIII         GATCTCACAGGCAATTCGGGTAGATACAATTTTGGTCGATTATTTAAAAATCTAAGTAAC
Conservation  ************************************************************

Bg273                                                                          1620
I             ACAATTTCGGTAAAACAATAATT-TATAATCCGTAAAAATTTTTGAGTGTGATTCGAAAT
II            ACAATTTCGGTAAAACAATAATT-TATAATCCGTAAAAATTTTTGAGTGTGATTCGAAAT
III           ACAATTTCGGTAAAACAATAATT-TATAATCCGTAAAAATTTTTGAGTGTGATTCGAAAT
IV            ACAATTTCGGTAAAACAATAATT-TATAATCCGTAAAAATTTTTGAGTGTGATTCGAAAT
V             ACAATTTCGGTAAAACAATAATTGTATAATCCGTAAAAATTTTTGAGTGTGATTCGAAAT
VI            ACAATTTCGGTAAAACAATAATTGTATAATCCGTAAAAATTTTTGAGTGTGATTCGAAAT
VII           ACAATTTCGGTAAAACAATAATTGTATAATCCGTAAAAATTTTTGAGTGTGATTCGAAAT
VIII          ACAATTTCGGTAAAACAATAATTGTATAATCCGTAAAAATTTTTGAGTGTGATTCGAAAT
IX            ACAATTTCGGTAAAACAATAATTGTATAATCCGTAAAAATTTTTGAGTGTGATTCGAAAT
X             ACAATTTCGGTAAAACAATAATTGTATAATCCGTAAAAACTTTTGAGTGTGATTCGAAAT
XI            ACAATTTCGGTAAAACAATAATTGTATAATCCGTAAAAACTTTTGAGTGTGATTCGAAAT
XII           ACAATTTCGGTAAAACAATAATT-TATAATCCGTAAAAATTTTTGAGTGTGATTCGAAAT
XIII          ACAATTTCGGTAAAACAATAATTGTATAATCCGTAAAAATTTTTGAGTGTGATTCGAAAT
XIV           ACAATTTCGGTAAAACAATAATTGTATAATCCGTAAAAATTTTTGAGTGTGATTCGAAAT
XV            ACAATTTCGGTAAAACAATAATTGTATAATCCGTAAAAATTTTTGAGTGTGATTCGAAAT
XVI           ACAATTTCGGTAAAACAATAATTGTATAATCCGTAAAAATTTTTGAGTGTGATTCGAAAT
XVII          ACAATTTCGGTAAAACAATAATTGTATAATCCGTAAAAACTTTTGAGTGTGATTCGAAAT
XVIII         ACAATTTCGGTAAAACAATAATTGTATAATCCGTAAAAACTTTTGAGTGTGATTCGAAAT
Conservation  *********************** *************** ********************

Bg273                                                                          1680
I             CTGTATGTAAAGGTTATTTATTTATTTATTTATTTATTTTTTTTAAAAAAAAGAAAAAAG
II            CTGTATGTAAAGGTTATTTATTTATTTATTTATTTATTTTTTTTAAAAAAAAGAAAAAAG
III           CTGTATGTAAAGGTTATTTATTTATTTATTTATTTATTTTTTTTAAAAAAAAGAAAAAAG
IV            CTGTATGTAAAGGTTATTTATTTATTTATTTATTTATTTTTTTTAAAAAAAAGAAAAAAG
V             CTGTATGTAAAGGTTATTTATTTATTTATTTATTTATTTTTTTAAAAAAAAAGAAAAAAG
VI            CTGTATGTAAAGGTTATTTATTTATTTATTTATTTATTTTTTTAAAAAAAAAGAAAAAAG
VII           CTGTATGTAAAGGTTATTTATTTATTTATTTATTTATTTTTTTAAAAAAAAAGAAAAAAG
VIII          CTGTATGTAAAGGTTATTTATTTATTTATTTATTTATTTTTTTAAAAAAAAAGAAAAAAG
IX            CTGTATGTAAAGGTTATTTATTTATTTATTTATTTATTTTTTTAAAAAAAAAGAAAAAAG
X             CTGTATGTAAAGGTTATTTATTTATTTATTTATTT-----TTTTAAAAAAAAGAAAAAAG
XI            CTGTATGTAAAGGTTATTTATTTATTTATTTATTT-----TTTTAAAAAAAAGAAAAAAG
XII           CTGTATGTAAAGGTTATTTATTTATTTATTTATTTATTTTTTTTAAAAAAAAGAAAAAAG
XIII          CTGTATGTAAAGGTTATTTATTTATTTATTTATTTATTTTTTTAAAAAAAAAGAAAAAAG
XIV           CTGTATGTAAAGGTTATTTATTTATTTATTTATTTATTTTTTTAAAAAAAAAGAAAAAAG
XV            CTGTATGTAAAG-TTATTTATTCATTTATTTATTTATTT-TTTAAAAAAAAAGAAAAAAA
XVI           CTGTATGTAAAG-TTATTTATTCATTTATTTATTTATTT-TT-AAAAAAAAAGAAAAAAA
XVII          CTGTATGTAAAGGTTATTTATTTATTTATTTATTT-----TTTTAAAAAAAAGAAAAAAG
XVIII         CTGTATGTAAAGGTTATTTATTTATTTATTTATTT-----TTTTAAAAAAAAGAAAAAAG
Conservation  ************ ********* ************     **  *************** 
```

```
             Bg273                                                                1740
I            AATAATCCTCTGTGTATTTATTTGAATCATTACTATTTTATTTCATTTTACTCTGAATTT
II           AATAATCCTCTGTGTATTTATTTGAATCATTACTATTTTATTTCATTTTACTCTGAATTT
III          AATAATCCTCTGTGTATTTATTTGAATCATTACTATTTTATTTCATTTTACTCTGAATTT
IV           AATAATCCTCTGTGTATTTATTTGAATCATTACTATTTTATTTCATTTTACTCTGAATTT
V            AATAATCCTCTGTGTATTTATTTGAATCATTACTATTTTATTTCATTTTACTCTGAATTT
VI           AATAATCCTCTGTGTATTTATTTGAATCATTACTATTTTATTTCATTTTACTCTGAATTT
VII          AATAATCCTCTGTGTATTTATTTGAATCATTACTATTTTATTTCATTTTACTCTGAATTT
VIII         AATAATCCTCTGTGTATTTATTTGAATCATTACTATTTTATTTCATTTTACTCTGAATTT
IX           AATAATCCTCTGTGTATTTATTTGAATCATTACTATTTTATTTCATTTTACTCTGAATTT
X            AATAATCCTCTGTGTATTTATTTGAATCATTACTATTTTATTTCATTTTACTCTGAATTT
XI           AATAATCCTCTGTGTATTTATTTGAATCATTACTATTTTATTTCATTTTACTCTGAATTT
XII          AATAATCCTCTGTGTATTTATTTGAATCATTACTATTTTATTTCATTTTACTCTGAATTT
XIII         AATAATCCTCTGTGTATTTATTTGAATCATTACTATTTTATTTCATTTTACTCTGAATTT
XIV          AATAATCCTCTGTGTATTTATTTGAATCATTACTATTTTATTTCATTTTACTCTGAATTT
XV           AAAAATCCTCTGTGTATTTATTTGAATCATTACTATTTTATTTCATTTTACTCTGAATTT
XVI          AAAAATCCTCTGTGTATTTATTTGAATCATTACTATTTTATTTCATTTTACTCTGAATTT
XVII         AATAATCCTCTGTGTATTTATTTGAATCATTACTATTTTATTTCATTTTACTCTGAATTT
XVIII        AATAATCCTCTGTGTATTTATTTGAATCATTACTATTTTATTTCATTTTACTCTGAATTT
Conservation ** *********************************************************

             Bg273                                                                1800
I            GCTTAGTTATGCCAACGGAATGCAAGATTTTAAAGGCAAAGCTTAGATACAATTATTAAT
II           GCTTAGTTATGCCAACGGAATGCAAGATTTTAAAGGCAAAGCTTAGATACAATTATTAAT
III          GCTTAGTTATGCCAACGGAATGCAAGATTTTAAAGGCAAAGCTTAGATACAATTATTAAT
IV           GCTTAGTTATGCCAACGGAATGCAAGATTTTAAAGGCAAAGCTTAGATACAATTATTAAT
V            GCTTAGTTATGCCAACGGAATGCAAGATTTTAAAGGCAAAGCTTAGATACAATTATTAAT
VI           GCTTAGTTATGCCAACGGAATGCAAGATTTTAAAGGCAAAGCTTAGATACAATTATTAAT
VII          GCTTAGTTATGCCAACGGAATGCAAGATTTTAAAGGCAAAGCTTAGATACAATTATTAAT
VIII         GCTTAGTTATGCCAACGGAATGCAAGATTTTAAAGGCAAAGCTTAGATACAATTATTAAT
IX           GCTTAGTTATGCCAACGGAATGCAAGATTTTAAAGGCAAAGCTTAGATACAATTATTAAT
X            GCTTAGTTATGCCAACGGAATGCAAGATTTTAAAGGCAAAGCTTAGATACAATTATTAAT
XI           GCTTAGTTATGCCAACGGAATGCAAGATTTTAAAGGCAAAGCTTAGATACAATTATTAAT
XII          GCTTAGTTATGCCAACGGAATGCAAGATTTTAAAGGCAAAGCTTAGATACAATTATTAAT
XIII         GCTTAGTTATGCCAACGGAATGCAAGATTTTAAAGGCAAAGCTTAGATACAATTATTAAT
XIV          GCTTAGTTATGCCAACGGAATGCAAGATTTTAAAGGCAAAGCTTAGATACAATTATTAAT
XV           GCTTAGTTATGCCAACGGAATGCAAGATTTTAAAGGCAAAGCTTAGATACAATTATTAAT
XVI          GCTTAGTTATGCCAACGGAATGCAAGATTTTAAAGGCAAAGCTTAGATACAATTATTAAT
XVII         GCTTAGTTATGCCAACGGAATGCAAGATTTTAAAGGCAAAGCTTAGATACAATTATTAAT
XVIII        GCTTAGTTATGCCAACGGAATGCAAGATTTTAAAGGCAAAGCTTAGATACAATTATTAAT
Conservation ************************************************************

             Bg276                                                                1860
I            TTTTTGAATATTCACGTTTATTATCAAATCAATAAAAAATGAAATTCGTTGCAACCCCTT
II           TTTTTGAATATTCACGTTTATTATCAAATCAATAAAAAATGAAATTCGTTGCAACCCCTT
III          TTTTTGAATATTCACGTTTATTATCAAATCAATAAAAAATGAAATTCGTTGCAACCC-TT
IV           TTTTTGAATATTCACGTTTATTATCAAATCAATAAAAAATGAAATTCGTTGCAACCCCTT
V            TTTTTGAATATTCACGTTTATTATCAAATCAATAAAAAATGAAATTCGTTGCAACCCCTT
VI           TTTTTGAATATTCACGTTTATTATCAAATCAATAAAAAATGAAATTCGTTGCAACCC-TT
VII          TTTTTGAATATTCACGTTTATTATCAAATCAATAAAAAATGAAATTCGTTGCAACCC-TT
VIII         TTTTTGAATATTCACGTTTATTATCAAATCAATAAAAAATGAAATTCGTTGCAACCCCTT
IX           TTTTTGAATATTCACGTTTATTATCAAATCAATAAAAAATGAAATTCGTTGCAACCCCTT
X            TTTTTGAATATTCACGTTTATTATCAAATCAATAAAAAATGAAATTCGTTGCAACCCCTT
XI           TTTTTGAATATTCACGTTTATTATCAAATCAATAAAAAATGAAATTCGTTGCAACCCCTT
XII          TTTTTGAATATTCACGTTTATTATCAAATCAATAAAAAATGAAATTCGTTGCAACCC-TT
XIII         TTTTTGAATATTCACGTTTATTATCAAATCAATAAAAAATGAAATTCGTTGCAACCC-TT
XIV          TTTTTGAATATTCACGTTTATTATCAAATCAATAAAAAATGAAATTCGTTGCAACCCCTT
XV           TTTTTGAATATTCACGTTTATTATCAAATCAATAAAA--TGAAATTCGTTGCAACCC-TT
XVI          TTTTTGAATATTCACGTTTATTATCAAATCAATAAAA--TGAAATTCGTTGCAACCC-TT
XVII         TTTTTGAATATTCACGTTTATTATCAAATCAATAAAAAATGAAATTCGTTGCAACCCCTT
XVIII        TTTTTGAATATTCACGTTTATTATCAAATCAATAAAAAATGAAATTCGTTGCAACCCCTT
Conservation *************************************  ****************** **

             Bg276                                                                1920
I            CAATATCACGTAATACTATTAAAAATTTTATTTTCTAACTTAGTATAAATAACATAACTA
II           CAATATCACGTAATACTATTAAAAATTTTATTTTCTAACTTAGTATAAATAACATAACTA
III          CAATATCACGTAAT--------------------CTAACTTAGTATAAATAACATAACTA
IV           CAATATCACGTAATACTATTAAAAATTTTATTTTCTAACTTAGTATAAATAACATAACTA
V            CAATATCACGTAATACTATTAAAAATTTTATTTTCTAACTTAGTATAAATAACATAACTA
VI           CAATATCACGTAAT--------------------CTAACTTAGTATAAATAACATAACTA
VII          CAATATCACGTAAT--------------------CTAACTTAGTATAAATAACATAACTA
VIII         CAATATCACGTAATACTATTAAAAATTTTATTTTCTAACTTAGTATAAATAACATAACTA
IX           CAATATCACGTAATACTATTAAAAATTTTATTTTCTAACTTAGTATAAATAACATAACTA
X            CAATATCACGTAATACTATTAAAAATTTTATTTTCTAACTTAGTATAAATAACATAACTA
XI           CAATATCACGTAATACTATTAAAAATTTTATTTTCTAACTTAGTATAAATAACATAACTA
XII          CAATATCACGTAAT--------------------CTAACTTAGTATAAATAACATAACTA
XIII         CAATATCACGTAAT--------------------CTAACTTAGTATAAATAACATAACTA
XIV          CAATATCACGTAATACTATTAAAAATTTTATTTTCTAACTTAGTATAAATAACATAACTA
XV           CAATATCACGTAATACTATTAAAAA---------CTAACTTAGTACAAATAACATAACTA
XVI          CAATATCACGTAATACTATTAAAAA---------CTAACTTAGTACAAATAACATAACTA
XVII         CAATATCACGTAAT--------------------CTAACTTAGTATAAATAACATAACTA
XVIII        CAATATCACGTAATACTATTAAACATTTTATTTTCTAACTTAGTATAAATAACATAACTT
Conservation **************                    *********** ************* 
```

```
Bg276                                                                            1980
I             TTATTTTCAATATTTTAATAT-----GTATAAATAATAATAATGATAAGAATAAAATTCA
II            TTATTTTCAATATTTTAATAT-----GTATAAATAATAATAATGATAAGAATAAAATTCA
III           TTATTTTCAATAT-------------GTATAAATAATAATAATGATAAGAATAAAATTCA
IV            TTATTTTCAATATTTTAATAT-----GTATAAATAATAATAATGATAAGAATAAAATTCA
V             TTATTTTCAATATTTTAATAT-----GTATAAATAATAATAATGATAAGAATAAAATTCA
VI            TTATTTTCAATAT-------------GTATAAATAATAATAATGATAAGAATAAAATTCA
VII           TTATTTTCAATAT-------------GTATAAATAATAATAATGATAAGAATAAAATTCA
VIII          TTATTTTCAATATTTTAATAT-----GTATAAATAATAATAATGATAAGAATAAAATTCA
IX            TTATTTTCAATATTTTAATAT-----GTATAAATAATAATAATGATAAGAATAAAATTCA
X             TTATTTTCAATATTTTAATAT-----GTATAAATAATAATAATGATAAGAATAAAATTCA
XI            TTATTTTCAATATTTTAATATAATTTGTACAAATAATAATAATGATAAGAATAAAATTCA
XII           TTATTTTCAATAT-------------GTATAAATAATAATAATGATAAGAATAAAATTCA
XIII          TTATTTTCAATAT-------------GTATAAATAATAATAATGATAAGAATAAAATTCA
XIV           TTATTTTCAATATTTTAATAT-----GTATAAATAATAATAATGATAAGAATAAAATTCA
XV            TTATTTTCAATAT-------------GTATAAATAATAATAATGATAAGAATAAAATTCA
XVI           TTATTTTCAATAT-------------GTATAAATAATAATAATGATAAGAATAAAATTCA
XVII          TTATTTTCAATAT-------------GTGTAAATAATAATAATGATAAGAATAAAAATCA
XVIII         TTATTTTCAATAT-------------GTATAAATAATAATAATGATAAGAATAAAATTCA
Conservation  *************             **  ************************** ***

Bg276                                                                            2040
I             AGTCGTAGAATTTGTCTAATTTTTTCGATGAGGTTGAATTCGAAAGCGACCAGGATATTT
II            AGTCGTAGAATTTGTCTAATTTTTTCGATGAGGTTGAATTCGAAAGCGACCAGGATATTT
III           AGTCGTAGAATTTGTCTAATTTTTTCGATGAGGTTGAATTCGAAAGCGACCAGGATATTT
IV            AGTCGTAGAATTTGTCTAATTTTTTCGATGAGGTTGAATTCGAAAGCGACCAGGATATTT
V             AGTCGTAGAATTTGTCTAATTTTTTCGATGAGGTTGAATTCGAAAGCGACCAGGATATTT
VI            AGTCGTAGAATTTGTCTAATTTTTTCGATGAGGTTGAATTCGAAAGCGACCAGGATATTT
VII           AGTCGTAGAATTTGTCTAATTTTTTCGATGAGGTTGAATTCGAAAGCGACCAGGATATTT
VIII          AGTCGTAGAATTTGTCTAATTTTTTCGATGAGGTTGAATTCGAAAGCGACCAGGATATTT
IX            AGTCGTAGAATTTGTCTAATTTTTTCGATGAGGTTGAATTCGAAAGCGACCAGGATATTT
X             AGTCGTAGAATTTGTCTAATTTTTTCGATGAGGTTGAATTCGAAAGCGACCAGGATATTT
XI            AGTCGTAGAATTTGTCTAATTTTTTCGATGAGGTTGAATTCGAAAGCGACCAGGATATTT
XII           AGTCGTAGAATTTGTCTAATTTTTTCGATGAGGTTGAATTCGAAAGCGACCAGGATATTT
XIII          AGTCGTAGAATTTGTCTAATTTTTTCGATGAGGTTGAATTCGAAAGCGACCAGGATATTT
XIV           AGTCGTAGAATTTGTCTAATTTTTTCGATGAGGTTGAATTCGAAAGCGACCAGGATATTT
XV            AGTCGTAGAATTTGTCTAATTTTTTCGATGAGGTTGAATTCGAAAGCGACCAGGATATTT
XVI           AGTCGTAGAATTTGTCTAATTTTTTCGATGAGGTTGAATTCGAAAGCGACCAGGATATTT
XVII          AGTCGTAGAATTTGTCTAATTTTTTCGATGAGGTTGAATTCGAAAGCGACCAGGATATTT
XVIII         AGTCGTAGAATTTGTCTAACTTTTTCGATGAGGTTGAATTCGAAAGCGACCAGGATATTT
Conservation  ******************* ****************************************

Bg276                                                                            2100
I             TAATGATACTACCGGATAAAGATTTGAAGCAAGTTTACCATCGGCACTATCTGGATATCC
II            TAATGATACTACCGGATAAAGATTTGAAGCAAGTTTACCATCGGCACTATCTGGATATCC
III           TAATGATACTACCGGATAAAGATTTGAAGCAAGTTTACCATCGGCACTATCTGGATATCC
IV            TAATGATACTACCGGATAAAGATTTGAAGCAAGTTTACCATCGGCACTATCTGGATATCC
V             TAATGATACTACCGGATAAAGATTTGAAGCAAGTTTACCATCGGCACTATCTGGATATCC
VI            TAATGATACTACCGGATAAAGATTTGAAGCAAGTTTACCATCGGCACTATCTGGATATCC
VII           TAATGATACTACCGGATAAAGATTTGAAGCAAGTTTACCATCGGCACTATCTGGATATCC
VIII          TAATGATACTACCGGATAAAGATTTGAAGCAAGTTTACCATCGGCACTATCTGGATATCC
IX            TAATGATACTACCGGATAAAGATTTGAAGCAAGTTTACCATCGGCACTATCTGGATATCC
X             TAATGATACTACCGGATAAAGATTTGAAGCAAGTTTACCATCGGCACTATCTGGATATCC
XI            TAATGATACTACCGGATAAAGATTTGAAGCAAGTTTACCATCGGCACTATCTGGATATCC
XII           TAATGATACTACCGGATAAAGATTTGAAGCAAGTTTACCATCGGCACTATCTGGATATCC
XIII          TAATGATACTACCGGATAAAGATTTGAAGCAAGTTTACCATCGGCACTATCTGGATATCC
XIV           TAATGATACTACCGGATAAAGATTTGAAGCAAGTTTACCATCGGCACTATCTGGATATCC
XV            TAATGATACTACCGGATAAAGATTTGAAGCAAGTTTACCATCGGCACTATCTGGATATCC
XVI           TAATGATACTACCGGATAAAGATTTGAAGCAAGTTTACCATCGGCACTATCTGGATATCC
XVII          TAATGATACTACCGGATAAAGATTTGAAGCAAGTTTACCATCGGCACTATCTGGATATCC
XVIII         TAATGATACTACCGGATAAAGATTTGAAGCAAGTTTACCATCGGCACTATCTGGATATCC
Conservation  ************************************************************

Bg303                                                                            2160
I             TAGTAAGTGACTTTATTTTATTTTATTTTATTTTATTTTTGATGATTAATAACAATCCAT
II            TAGTAAGTGACTTTATTTTATTTTATTTTATTTTATTTTTGATGATTAATAACAATCCAT
III           TAGTAAGTGACTTTATTTTATTTTATTTTATTTTATTTTTGATGATTAATAACAATCCAT
IV            TAGTAAGTGACTTTATTTTATTTTATTTTATTTTATTTTTGATGATTAATAACAATCCAT
V             TAGTAAGTGACTTTATTTTATTTTATTTTATTTTATTTTTGATGATTAATAACAATCCAT
VI            TAGTAAGTGACTTTATTTTATTTTATTTTATTTTATTTTTGATGATTAATAACAATCCAT
VII           TAGTAAGTGACTTTATTTTATTTTATTTTATTTTATTTTTGATGATTAATAACAATCCAT
VIII          TAGTAAGTGACTTTATTTTATTTTATTTTATTTTATTTTTGATGATTAATAACAATCCAT
IX            TAGTAAGTGACTTTATTTTATTTTATTTTATTTTATTTTTGATGATTAATAACAATCCAT
X             TAGTAAGTGACTTTATTTTATTTTATTTTATTTTATTTTTGATGATTAATAACAATCCAT
XI            TAGTAAGTGACTTTATTTTATTTTATTTTATTTTATTTTTGATGATTAATAACAATCCAT
XII           TAGTAAGTGACTTTATTTTATTTTATTTTATTTTATTTTTGATGATTAATAACAATCCAT
XIII          TAGTAAGTGACTTTATTTTATTTTATTTTATTTTATTTTTGATGATTAATAACAATCCAT
XIV           TAGTAAGTGACTTTATTTTATTTTATTTTATTTTATTTTTGATGATTAATAACAATCCAT
XV            TAGTAAGTGACTTTATTTTATTTTATTTTATTTTATTTTTGATGATTAATAACAATCCAT
XVI           TAGTAAGTGACTTTATTTTATTTTATTTTATTTTATTTTTGATGATTAATAACAATCCAT
XVII          TAGTAAGTGACT--ATTTTATTTTATTTTATTTTATTTTTGATGATTAATAACAATCCAT
XVIII         TAGTAAGTGACT--ATTTTATTTTATTTTATTTTATTTTTGATGATTAATAACAATCCAT
Conservation  ************  **********************************************
```

```
Bg303                                                                     2220
I             CACATATAAAGAATTAATTAAACTTCAAGATAATTGTTAGTCTCATCAAACGTCCAATCA
II            CACATATAAAGAATTAATTAAACTTCAAGATAATTGTTAGTCTCATCAAACGTCCAATCA
III           CACATATAAAGAATTAATTAAACTTCAAGATAATTGTTAGTCTCATCAAACGTCCAATCA
IV            CACATATAAAGAATTAATTAAACTTCAAGATAATTGTTAGTCTCATCAAACGTCCAATCA
V             CACATATAAAGAATTAATTAAACTTCAAGATAATTGTTAGTCTCATCAAACGTCCAATCA
VI            CACATATAAAGAATTAATTAAACTTCAAGATAATTGTTAGTCTCATCAAACGTCCAATCA
VII           CACATATAAAGAATTAATTAAACTTCAAGATAATTGTTAGTCTCATCAAACGTCCAATCA
VIII          CACATATAAAGAATTAATTAAACTTCAAGATAATTGTTAGTCTCATCAAACGTCCAATCA
IX            CACATATAAAGAATTAATTAAACTTCAAGATAATTGTTAGTCTCATCAAACGTCCAATCA
X             CACATATAAAGAATTAATTAAACTTCAAGATAATTGTTAGTCTCATCAAACGTCCAATCA
XI            CACATATAAAGAATTAATTAAACTTCAAGATAATTGTTAGTCTCATCAAACGTCCAATCA
XII           CACATATAAAGAATTAATTAAACTTCAAGATAATTGTTAGTCTCATCAAACGTCCAATCA
XIII          CACATATAAAGAATTAATTAAACTTCAAGATAATTGTTAGTCTCATCAAACGTCCAATCA
XIV           CACATATAAAGAATTAATTAAACTTCAAGATAATTGTTAGTCTCATCAAACGTCCAATCA
XV            CACATATAAAGAATTAATTAAACTTCAAGATAATTGTTAGTCTCATCAAACGTCCAATCA
XVI           CACATATAAAGAATTAATTAAACTTCAAGATAATTGTTAGTCTCATCAAACGTCCAATCA
XVII          CACATATAA-GAATTAATTAAACTTCAAGATAATTGTTAGTCTCATCAAACGTCCAATCA
XVIII         CACATATAA-GAATTAATTAAACTTCAAGATAATTGTTAGTCTCATCAAACGTCCAATCA
Conservation  ********* **************************************************
```

```
Bg348                                                                     2280
I             TATTTTGCGGAGAATAAAATGTGTATGAAGCACAATCTGCGTAAAAAAAAA-TAATAATA
II            TATTTTGCGGAGAATAAAATGTGTATGAAGCACAATCTGCGTAAAAAAAAA-TAATAATA
III           TATTTTGCGGAGAATAAAATGTGTATGAAGCACAATCTGCGTAAAAAAAAA-TAATAATA
IV            TATTTTGCGGAGAATAAAATGTGTATGAAGCACAATCTGCGTAAAAAAAAA-TAATAATA
V             TATTTTGCGGAGAATAAAATGTGTATGAAGCACAATCTGCGTAAAAAAAAA-TAATAATA
VI            TATTTTGCGGAGAATAAAATGTGTATGAAGCACAATCTGCGTAAAAAAAAA-TAATAATA
VII           TATTTTGCGGAGAATAAAATGTGTATGAAGCACAATCTGCGTAAAAAAAAA-TAATAATA
VIII          TATTTTGCGGAGAATAAAATGTGTATGAAGCACAATCTGCGTAAAAAAAAA-TAATAATA
IX            TATTTTGCGGAGAATAAAATGTGTATGAAGCACAATCTGCGTAAAAAAAAAATAATAATA
X             TATTTTGCGGAGAATAAAATGTGTATGAAGCACAATCTGCGTAAAAAAAAA-TAATAATA
XI            TATTTTGCGGAGAATAAAATGTGTATGAAGCACAATCTGCGTAAAAAAAAA-TAATAATA
XII           TATTTTGCGGAGAATAAAATGTGTATGAAGCACAATCTGCGTAAAAAAAAA-TAATAATA
XIII          TATTTTGCGGAGAATAAAATGTGTATGAAGCACAATCTGCGTAAAAAAAAA-TAATAATA
XIV           TATTTTGCGGAGAATAAAATGTGTATGAAGCACAATCTGCGTAAAAAAAAA-TAATAATA
XV            TATTTTGCGGAGAATAAAATGTGTATGAAGCACAATCTGCGTAAAAAAAAA-TAATAATA
XVI           TATTTTGCGGAGAATAAAATGTGTATGAAGCACAATCTGCGTAAAAAAAAA-TAATAATA
XVII          TATTTTGCGGAGAATAAAATGTGTATGAAGCACAATCTGCGTAAAAAAAAAATAATAATA
XVIII         TATTTTGCGGAGAATAAAATGTGTATGAAGCACAATCTGCGTAAAAAAAAA-TAATAATA
Conservation  *************************************************** ********
```

```
Bg348                                                                     2340
I             AATAAATAAATAAAAATA------AAATAAAATAAA-CAAATAAATAAAAATCATCTTTA
II            AATAAATAAATAAAAATA------AAATAAAATAAA-CAAATAAATAAAAATCATCTTTA
III           AATAAATAAATAAAAATA------AAATAAAATAAA-CAAATAAATAAAAATCATCTTTA
IV            AATAAATAAATAAAAATA------AAATAAAATAAA-CAAATAAATAAAAATCATCTTTA
V             AATAAATAAATAAAAATA------AAATAAAATAAA-CAAATAAATAAAAATCATCTTTA
VI            AATAAATAAATAAAAATA------AAATAAAATAAA-CAAATAAATAAAAATCATCTTTA
VII           AATAAATAAATAAAAATA------AAATAAAATAAA-CAAATAAATAAAAATCATCTTTA
VIII          AATAAATAAATAAAAATA------AAATAAAATAAA-CAAATAAATAAAAATCATCTTTA
IX            AATAAATAAATAAATAAATAATGTAAATAAAATAAAATAAACAAATAAAAATCATCTTT-
X             AATAAATAAATAAAAATA------AAATAAAATAAA-CAAATAAATAAAAATCATCTTTA
XI            AATAAATAAATAAAAATA------AAATAAAATAAA-CAAATAAATAAAAATCATCTTTA
XII           AATAAATAAATAAAAATA------AAATAAAATAAA-CAAATAAATAAAAATCATCTTTA
XIII          AATAAATAAATAAAAATA------AAATAAAATAAA-CAAATAAATAAAAATCATCTTTA
XIV           AATAAATAAATAAAAATA------AAATAAAATAAA-CAAATAAATAAAAATCATCTTTA
XV            A-TAAATAAATAAAAATA------AAATAAAATAAA-CAAATAAATAAAAATCATCTT-A
XVI           A-TAAATAAATAAAAATA------AAATAAAATAAA-CAAATAAATAAAAATCATCTT-A
XVII          AATAAATAAATAAATAAATAATGTAAATAAAATAAAATAAACAAATAAAAATCATCTTT-
XVIII         AATAAATAAATAAATAAAT----------AAATAAAATAAACAAATAAAAATCATCTT-A
Conservation  * ************ * *           *******  *** ****************
```

```
Bg348                                                                     2400
I             TAAAAGTAATTTTAATTTACCCGTTCCAACAATCGTCCATACTGCGGCATCTGTAACATC
II            TAAAAGTAATTTTAATTTACCCGTTCCAACAATCGTCCATACTGCGGCATCTGTAACATC
III           TAAAAGTAATTTTAATTTACCCGTTCCAACAATCGTCCATACTGCGGCATCTGTAACATC
IV            TAAAAGTAATTTTAATTTACCCGTTCCAACAATCGTCCATACTGCGGCATCTGTAACATC
V             TAAAAGTAATTTTAATTTACCCGTTCCAACAATCGTCCATACTGCGGCATCTGTAACATC
VI            TAAAAGTAATTTTAATTTACCCGTTCCAACAATCGTCCATACTGCGGCATCTGTAACATC
VII           TAAAAGTAATTTTAATTTACCCGTTCCAACAATCGTCCATACTGCGGCATCTGTAACATC
VIII          TAAAAGTAATTTTAATTTACCCGTTCCAACAATCGTCCATACTGCGGCATCTGTAACATC
IX            TAAAAGTAATTTTAATTTACCCGTTCCAACAATCGTCCATACTGCGGCATCTGTAACATC
X             TAAAAGTAATTTTAATTTACCCGTTCCAACAATCGTCCATACTGCGGCATCTGTAACATC
XI            TAAAAGTAATTTTAATTTACCCGTTCCAACAATCGTCCATACTGCGGCATCTGTAACATC
XII           TAAAAGTAATTTTAATTTACCCGTTCCAACAATCGTCCATACTGCGGCATCTGTAACATC
XIII          TAAAAGTAATTTTAATTTACCCGTTCCAACAATCGTCCATACTGCGGCATCTGTAACATC
XIV           TAAAAGTAATTTTAATTTACCCGTTCCAACAATCGTCCATACTGCGGCATCTGTAACATC
XV            TAAAAGTAATTTTAATTTACCCGTTCCAACAATCGTCCATACTGCGGCATCTGTAACATC
XVI           TAAAAGTAATTTTAATTTACCCGTTCCAACAATCGTCCATACTGCGGCATCTGTAACATC
XVII          TAAAAGTAATTTTAATTTACCCGTTCCAACAATCGTCCATACTGCGGCATCTGTAACATC
XVIII         TAAAAGTAATTTTAATTTACCCGTTCCAACAATCGTCCATACTGCGGCATCTGTAACATC
Conservation  ************************************************************
```

```
Bg348                                                                          2460
I             TTCAGTCCATAAAGCTCCATTCCTCTTTAACAAAAATCATCTATCCTTTTTACGTAAATT
II            TTCAGTCCATAAAGCTCCATTCCTCTTTAACAAAAATCATCTATCCTTTTTACGTAAATT
III           TTCAGTCCATAAAGCTCCATTCCTCTTTAACAAAAATCATCTATCCTTTTTACGTAAATT
IV            TTCAGTCCATAAAGCTCCATTCCTCTTTAACAAAAATCATCTATCCTTTTTACGTAAATT
V             TTCAGTCCATAAAGCTCCATTCCTCTTTAACAAAAATCATCTATCCTTTTTACGTAAATT
VI            TTCAGTCCATAAAGCTCCATTCCTCTTTAACAAAAATCATCTATCCTTTTTACGTAAATT
VII           TTCAGTCCATAAAGCTCCATTCCTCTTTAACAAAAATCATCTATCCTTTTTACGTAAATT
VIII          TTCAGTCCATAAAGCTCCATTCCTCTTTAACAAAAATCATCTATCCTTTTTACGTAAATT
IX            TTCAGTCCATAAAGCTCCATTCCTCTTTAACAAAAATCATCTATCCTTTTTACGTAAATT
X             TTCAGTCCATAAAGCTCCATTCCTCTTTAACAAAAATCATCTATCCTTTTTACGTAAATT
XI            TTCAGTCCATAAAGCTCCATTCCTCTTTAACAAAAATCATCTATCCTTTTTACGTAAATT
XII           TTCAGTCCATAAAGCTCCATTCCTCTTTAACAAAAATCATCTATCCTTTTTACGTAAATT
XIII          TTCAGTCCATAAAGCTCCATTCCTCTTTAACAAAAATCATCTATCCTTTTTACGTAAATT
XIV           TTCAGTCCATAAAGCTCCATTCCTCTTTAACAAAAATCATCTATCCTTTTTACGTAAATT
XV            TTCAGTCCATAAAGCTCCATTCCTCTTTAACAAAAATCATCTATCCTTTTTACGTAAATT
XVI           TTCAGTCCATAAAGCTCCATTCCTCTTTAACAAAAATCATCTATCCTTTTTACGTAAATT
XVII          TTCAGTCCATAAAGCTCCATTCCTCTTTAACAAAAATCATCTATCCTTTTTACGTAAATT
XVIII         TTCAGTCCATAAAGCTCCATTCCTCTTTAACAAAAATCATCTATCCTTTTTACGTAAATT
Conservation  ************************************************************
```

```
Bg355                                                                          2520
I             ATATATAGTTTTCATATATTTTGAATATAATAGTATTTAAATGGTCCTCTCTTTTAACTT
II            ATATATAGTTTTCATATATTTTGAATATAATAGTATTTAAATGGTCCTCTCTTTTAACTT
III           ATATATAGTTTTCATATATTTTGAATATAATAGTATTTAAATGGTCCTCTCTTTTAACTT
IV            ATATATAGTTTTCATATATTTTGAATATAATAGTATTTAAATGGTCCTCTCTTTTAACTT
V             ATATATAGTTTTCATATATTTTGAATATAATAGTATTTAAATGGTCCTCTCTTTTAACTT
VI            ATATATAGTTTTCATATATTTTGAATATAATAGTATTTAAATGGTCCTCTCTTTTAACTT
VII           ATATATAGTTTTCATATATTTTGAATATAATAGTATTTAAATGGTCCTCTCTTTTAACTT
VIII          ATATATAGTTTTCATATATTTTGAATATAATAGTATTTAAATGGTCCTCTCTTTTAACTT
IX            ATATATAGTTTTCATATATTTTGAATATAATAGTATTTAAATGGTCCTCTCTTTTAACTT
X             ATATATAGTTTTCATATATTTTGAATATAATAGTATTTAAATGGTCCTCTCTTTTAACTT
XI            ATATATAGTTTTCATATATTTTGAATATAATAGTATTTAAATGGTCCTCTCTTTTAACTT
XII           ATATATAGTTTTCATATATTTTGAATATAATAGTATTTAAATGGTCCTCTCTTTTAACTT
XIII          ATATATAGTTTTCATATATTTTGAATATAATAGTATTTAAATGGTCCTCTCTTTTAACTT
XIV           ATATATAGTTTTCATATATTTTGAATATAATAGTATTTAAATGGTCCTCTCTTTTAACTT
XV            ATATATAGTTTTCATATATTTTGAATATAATAGTATTTAAATGGTCCTCTCTTTTAACTT
XVI           ATATATAGTTTTCATATATTTTGAATATAATAGTATTTAAATGGTCCTCTCTTTTAACTT
XVII          ATATATAGTTTTCATATATTTTGAATATCATAGTATTTAAATGGTCCTCTCTTTTAACTT
XVIII         ATATATAGTTTTCATATATTTTGAATATTATAGTATATAAATGGATCTCTCTTTTAACTT
Conservation  **************************** ******* *******  **************
```

```
Bg355                                                                          2580
I             CCAATCGGAATATAAAGTCTATCGTAATATAAGTTTATTTGTTTTTATGGTTTTCGTGAA
II            CCAATCGGAATATAAAGTCTATCGTAATATAAGTTTATTTGTTTTTATGGTTTTCGTGAA
III           CCAATCGGAATATAAAGTCTATCGTAATATAAGTTTATTTGTTTTTATGGTTTTCGTGAA
IV            CCAATCGGAATATAAAGTCTATCGTAATATAAGTTTATTTGTTTTTATGGTTTTCGTGAA
V             CCAATCGGAATATAAAGTCTATCGTAATATAAGTTTATTTGTTTTTATGGTTTTCGTGAA
VI            CCAATCGGAATATAAAGTCTATCGTAATATAAGTTTATTTGTTTTTATGGTTTTCGTGAA
VII           CCAATCGGAATATAAAGTCTATCGTAATATAAGTTTATTTGTTTTTATGGTTTTCGTGAA
VIII          CCAATCGGAATATAAAGTCTATCGTAATATAAGTTTATTTGTTTTTATGGTTTTCGTGAA
IX            CCAATCGGAATATAAAGTCTATCGTAATATAAGTTTATTTGTTTTTATGGTTTTCGTGAA
X             CCAATCGGAATATAAAGTCTATCGTAATATAAGTTTATTTGTTTTTATGGTTTTCGTGAA
XI            CCAATCGGAATATAAAGTCTATCGTAATATAAGTTTATTTGTTTTTATGGTTTTCGTGAA
XII           CCAATCGGAATATAAAGTCTATCGTAATATAAGTTTATTTGTTTTTATGGTTTTCGTGAA
XIII          CCAATCGGAATATAAAGTCTATCGTAATATAAGTTTATTTGTTTTTATGGTTTTCGTGAA
XIV           CCAATCGGAATATAAAGTCTATCGTAATATAAGTTTATTTGTTTTTATGGTTTTCGTGAA
XV            CCAATCGGAATATAAAGTCTATCGTAATATAAGTTTATTTGTTTTTATGGTTTTCGTGAA
XVI           CCAATCGGAATATAAAGTCTATCGTAATATAAGTTTATTTGTTTTTATGGTTTTCGTGAA
XVII          CCAATCGG-----------------AATATAAGTTTATTTGTTTTTATGGTTTTCGTGAA
XVIII         TCCA-----------ATCCTCTCGTAATATATCTTTATATGATTTTATGGTTTTCGTGAA
Conservation   * *                     ******  ***** ** ******************
```

```
Bg355                                                                          2640
I             TATACTATATAATAAACTAGATTGTATTCTCATGATCTTTCTCCTTTCAGTATATTTGAT
II            TATACTATATAATAAACTAGATTGTATTCTCATGATCTTTCTCCTTTCAGTATATTTGAT
III           TATACTATATAATAAACTAGATTGTATTCTCATGATCTTTCTCCTTTCAGTATATTTGAT
IV            TATACTATATAATAAACTAGATTGTATTCTCATGATCTTTCTCCTTTCAGTATATTTGAT
V             TATACTATATAATAAACTAGATTGTATTCTCATGATCTTTCTCCTTTCAGTATATTTGAT
VI            TATACTATATAATAAACTAGATTGTATTCTCATGATCTTTCTCCTTTCAGTATATTTGAT
VII           TATACTATATAATAAACTAGATTGTATTCTCATGATCTTTCTCCTTTCAGTATATTTGAT
VIII          TATACTATATAATAAACTAGATTGTATTCTCATGATCTTTCTCCTTTCAGTATATTTGAT
IX            TATACTATATAATAAACTAGATTGTATTCTCATGATCTTTCTCCTTTCAGTATATTTGAT
X             TATACTATATAATAAACTAGATTGTATTCTCATGATCTTTCTCCTTTCAGTATATTTGAT
XI            TATACTATATAATAAACTAGATTGTATTCTCATGATCTTTCTCCTTTCAGTATATTTGAT
XII           TATACTATATAATAAACTAGATTGTATTCTCATGATCTTTCTCCTTTCAGTATATTTGAT
XIII          TATACTATATAATAAACTAGATTGTATTCTCATGATCTTTCTCCTTTCAGTATATTTGAT
XIV           TATACTATATAATAAACTAGATTGTATTCTCATGATCTTTCTCCTTTCAGTATATTTGAT
XV            TATACTATATAATAAACTAGATTGTATTCTCATGATCTTTCTCCTTTCAGTATATTTGAT
XVI           TATACTATATAATAAACTAGATTGTATTCTCATGATCTTTCTCCTTTCAGTATATTTGAT
XVII          TATACTATATAATAAACTAGATTGTATTCTCATGATCTTTCTCCTTTCAGTATATTTGAT
XVIII         TATACTATATAATAAACTAGATTGTATTCTCATGATCTTTCTCCTTTCAGTATATATGAT
Conservation  ******************************************************* ****
```

```
Bg355                                                                        2700
I             CATCCTACTTTCTCTAGCGTAATTTTCAGAATATAATATGTATCATCTGATAATAGCTTG
II            CATCCTACTTTCTCTAGCGTAATTTTCAGAATATAATATGTATCATCTGATAATAGCTTG
III           CATCCTACTTTCTCTAGCGTAATTTTCAGAATATAATATGTATCATCTGATAATAGCTTG
IV            CATCCTACTTTCTCTAGCGTAATTTTCAGAATATAATATGTATCATCTGATAATAGCTTG
V             CATCCTACTTTCTCTAGCGTAATTTTCAGAATATAATATGTATCATCTGATAATAGCTTG
VI            CATCCTACTTTCTCTAGCGTAATTTTCAGAATATAATATGTATCATCTGATAATAGCTTG
VII           CATCCTACTTTCTCTAGCGTAATTTTCAGAATATAATATGTATCATCTGATAATAGCTTG
VIII          CATCCTACTTTCTCTAGCGTAATTTTCAGAATATAATATGTATCATCTGATAATAGCTTG
IX            CATCCTACTTTCTCTAGCGTAATTTTCAGAATATAATATGTATCATCTGATAATAGCTTG
X             CATCCTACTTTCTCTAGCGTAATTTTCAGAATATAATATGTATCATCTGATAATAGCTTG
XI            CATCCTACTTTCTCTAGCGTAATTTTCAGAATATAATATGTATCATCTGATAATAGCTTG
XII           CATCCTACTTTCTCTAGCGTAATTTTCAGAATATAATATGTATCATCTGATAATAGCTTG
XIII          CATCCTACTTTCTCTAGCGTAATTTTCAGAATATAATATGTATCATCTGATAATAGCTTG
XIV           CATCCTACTTTCTCTAGCGTAATTTTCAGAATATAATATGTATCATCTGATAATAGCTTG
XV            CATCCTACTTTCTCTAGCGTAATTTTCAGAATATAATATGTATCATCTGATAATAGCTTG
XVI           CATCCTACTTTCTCTAGCGTAATTTTCAGAATATAATATGTATCATCTGATAATAGCTTG
XVII          CATCCTACTTTCTCTAGCGTAATTTTCAGAATATAATATGTATCATCTGATAATAGCTTG
XVIII         CATCCTACTTTTTCTAGCGTAATTTTCAGAATATAATATGTATCATCTGATAATAGCTTG
Conservation  *********** ************************************************

Bg355                                                                        2760
I             TTTCCATTGAAATATACTCATAAAGTGATCGATAACATGTCAAAAAATTTTCAACGTCCA
II            TTTCCATTGAAATATACTCATAAAGTGATCGATAACATGTCAAAAAATTTTCAACGTCCA
III           TTTCCATTGAAATATACTCATAAAGTGATCGATAACATGTCAAAAAATTTTCAACGTCCA
IV            TTTCCATTGAAATATACTCATAAAGTGATCGATAACATGTCAAAAAATTTTCAACGTCCA
V             TTTCCATTGAAATATACTCATAAAGTGATCGATAACATGTCAAAAAATTTTCAACGTCCA
VI            TTTCCATTGAAATATACTCATAAAGTGATCGATAACATGTCAAAAAATTTTCAACGTCCA
VII           TTTCCATTGAAATATACTCATAAAGTGATCGATAACATGTCAAAAAATTTTCAACGTCCA
VIII          TTTCCATTGAAATATACTCATAAAGTGATCGATAACATGTCAAAAAATTTTCAACGTCCA
IX            TTTCCATTGAAATATACTCATAAAGTGATCGATAACATGTCAAAAAATTTTCAACGTCCA
X             TTTCCATTGAAATATACTCATAAAGTGATCGATAACATGTCAAAAAATTTTCAACGTCCA
XI            TTTCCATTGAAATATACTCATAAAGTGATCGATAACATGTCAAAAAATTTTCAACGTCCA
XII           TTTCCATTGAAATATACTCATAAAGTGATCGATAACATGTCAAAAAATTTTCAACGTCCA
XIII          TTTCCATTGAAATATACTCATAAAGTGATCGATAACATGTCAAAAAATTTTCAACGTCCA
XIV           TTTCCATTGAAATATACTCATAAAGTGATCGATAACATGTCAAAAAATTTTCAACGTCCA
XV            TTTCCATTGAAATATACTCATAAAGTGATCGATAACATGTCAAAAAATTTTCAACGTCCA
XVI           TTTCCATTGAAATATACTCATAAAGTGATCGATAACATGTCAAAAAATTTTCAACGTCCA
XVII          TTTCCATTGAAATATACTCATAAAGTGATCGATAACATGTCAAAAAATTTTCAACGTCCA
XVIII         TTTCCATTGAAATATACTCATAAAGTGATCGATAACATGTCAAAAAATTTTCAACGTCCA
Conservation  ************************************************************

nr int                                                                       2820
I             TTTTTGTTTCTATTTATGATTTAATCTATTTTGA-TGAAA-TAATAATAAAAATGATTTA
II            TTTTTGTTTCTATTTATGATTTAATCTATTTTGA-TGAAA-TAATAATAAAAATGATTTA
III           TTTTTGTTTCTATTTATGATTTAATCTATTTTGA-TGAAA-TAATAATAAAAATGATTTA
IV            TTTTTGTTTCTATTTATGATTTAATCTATTTTGA-TGAAA-TAATAATAAAAATGATTTA
V             TTTTTGTTTCTATTTATGATTTAATCTATTTTGA-TGAAA-TAATAATAAAAATGATTTA
VI            TTTTTGTTTCTATTTATGATTTAATCTATTTTGA-TGAAA-TAATAATAAAAATGATTTA
VII           TTTTTGTTTCTATTTATGATTTAATCTATTTTGA-TGAAA-TAATAATAAAAATGATTTA
VIII          TTTTTGTTTCTATTTATGATTTAATCTATTTTGA-TGAAA-TAATAATAAAAATGATTTA
IX            TTTTTGTTTCTATTTATGATTTAATCTATTTTGA-TGAAA-TAATAATAAAAATGATTTA
X             TTTTTGTTTCTATTTATGATTTAATCTATTTTGA-TGAAA-TAATAATAAAAATGATTTA
XI            TTTTTGTTTCTATTTATGATTTAATCTATTTTGA-TGAAA-TAATAATAAAAATGATTTA
XII           TTTTTGTTTCTATTTATGATTTAATCTATTTTGA-TGAAAATAATAATAAAAATGATTTA
XIII          TTTTTGTTTCTATTTATGATTTAATCTATTTTGA-TGAAA-TAATAATAAAAATGATTTA
XIV           TTTTTGTTTCTATTTATGATTTAATCTATTTTGA-TGAAA-TAATAATAAAAATGATTTA
XV            TTTTTGTTTCTATTTATGATTTAATCTATTTTGA-TGAA--TAATAATAAAAATGATTTA
XVI           TTTTTGTTTCTATTTATGATTTAATCTATTTTGA-TGAA--TAATAATAAAAATGATTTA
XVII          TTTTTGTTTCTATTTATGATTTAATCTATTTTGAT---GAATAATAATAAAAATGATTTA
XVIII         TTTTTGTTTCTATTTATGATGTAATCTATTTTGAATGAAAATAATAATAAAAATGATTTA
Conservation  ******************** *************       *******************

nr int                                                                       2880
I             ATAAGTATTATATAATAATGATTATAATCTTTTTT-GTTTTCTTTTATAAGAAATTTTTT
II            ATAAGTATTATATAATAATGATTATAATCTTTTTT-GTTTTCTTTTATAAGAAATTTTTT
III           ATAAGTATTATATAATAATGATTATAATCTTTTTT-GTTTTCTTTTATAAGAAATTTTTT
IV            ATAAGTATTATATAATAATGATTATAATCTTTTTT-GTTTTCTTTTATAAGAAATTTTTT
V             ATAAGTATTATATAATAATGATTATAATCTTTTTT-GTTTTCTTTTATAAGAAATTTTTT
VI            ATAAGTATTATATAATAATGATTATAATCTTTTTT-GTTTTCTTTTATAAGAAATTTTTT
VII           ATAAGTATTATATAATAATGATTATAATCTTTTTT-GTTTTCTTTTATAAGAAATTTTTT
VIII          ATAAGTATTATATAATAATGATTATAATCTTTTTT-GTTTTCTTTTATAAGAAATTTTTT
IX            ATAAGTATTATATAATAATGATTATAATCTTTTTT-GTTTTCTTTTATAAGAAATTTTTT
X             ATAAGTATTATATAATAATGATTATAATCTTTTTT-GTTTTCTTTTATAAGAAATTTTTT
XI            ATAAGTATTATATAATAATGATTATAATCTTTTTT-GTTTTCTTTTATAAGAAATTTTTT
XII           ATAAGTATTATATAATAATGATTATAATCTTTTTT-GTTTTCTTTTATAAGAAATTTTTT
XIII          ATAAGTATTATATAATAATGATTATAATCTTTTTT-GTTTTCTTTTATAAGAAATTTTTT
XIV           ATAAGTATTATATAATAATGATTATAATCTTTTTT-GTTTTCTTTTATAAGAAATTTTTT
XV            ATAAGTATTATATAATAATGATTATAATCTTTTTT-GTTTTCTTTTATAAGAAATTTTTT
XVI           ATAAGTATTATATAATAATGATTATAATCTTTTTT-GTTTTCTTTTATAAGAAATTTTTT
XVII          ATAAGTATTATATAATAATGATTATAATCTTTTT--GTTTTCTTTTATAAGAAATTTTTT
XVIII         ATAAGTATTATATAATAATGATTATAATCTTTTTTTGTTTTCTTTTATAAGAAATTTTTT
Conservation  **********************************  ************************
```

```
nr int                                                                     2940
I             TGTTCTCTTTTTTTTTTTGATTATTGATTTAAAAAGAACAATAATAAAAAAAAAAAAAAA
II            TGTTCTCTTTTTTTTTTTGATTATTGATTTAAAAAGAACAATAATAAAAAAAAAAAAAAA
III           TGTTCTCTTTTTTTTTTTGATTATTGATTTAAAAAGAACAATAATAAAAAAAAAAAAAAA
IV            TGTTCTCTTTTTTTTTTTGATTATTGATTTAAAAAGAACAATAATAAT--AAAAAAAAAA
V             TGTTCTCTTTTTTTTTTTGATTATTGATTTAAAAAGAACAATAATAAT--AAAAAAAAAA
VI            TGTTCTCTTTTTTTTTTTGATTATTGATTTAAAAAGAACAATAATAAT--AAAAAAAAAA
VII           TGTTCTCTTTTTTTTTTTGATTATTGATTTAAAAAGAACAATAATAAT--AAAAAAAAAA
VIII          TGTTCTCTTTTTTTTTTTGATTATTGATTTAAAAAGAACAATAATAAT--AAAAAAAAAA
IX            TGTTCTCTTTTTTTTTTTGATTATTGATTTAAAAAGAACAATAATAAAAAAAAAAAAAAA
X             TGTTCTCTTTTTTTTTTTGATTATTGATTTAAAAAGAACAATAATAAT--AAAAAAAAAA
XI            TGTTCTCTTTTTTTTTTTGATTATTGATTTAAAAAGAACAATAATAAT--AAAAAAAAAA
XII           TGTTCTCTTTTTTTTTTTGATTATTGATTTAAAAAGAACAATAAAAAAAAAAAAAAAAAA
XIII          TGTTCTCTTTTTTTTTTTGATTATTGATTTAAAAAGAACAATAATAATAAAAAAAAAAAA
XIV           TGTTCTCTTTTTTTTTTTGATTATTGATTTAAAAAGAACAATAATAAT--AAAAAAAAAA
XV            TGTTCTCTTTTTTTTT--GATTATTGATTTAAAAAGAACAATAATAA-----AAAAAAAA
XVI           TGTTCTCTTTTTTTTT--GATTATTGATTTAAAAAGAACAATAATAA-----AAAAAAAA
XVII          TGTTCTCTTTTTTTTT--GATTATTGATTTAAAA-GAACAATAATAA-AAAAAAAAAAAA
XVIII         TGTTCTCTTTTTTTTT--GATTATTGATTTAAAA-GAACAATAATAAT----AAAAAAAA
Conservation  ****************  **************** ********* **     ********

nr int                                                                     3000
I             AAAAAAAAAATGAAAAAAAATGAAAAAATGAAAAA-----AAAAATTTGCTTTTTACTTA
II            AAAAAAAAAATGAAAAAAAATGAAAAAATGAAAAA-----AAAAATTTGCTTTTTACTTA
III           AAAAAAAAAATGAAAAAAAATGAAAAAATGAAAAA-----AAAAATTTGCTTTTTACTTA
IV            AAAAAAAAAATGAAAAAAAATGAAAAAATGAAAAA-----AAAAATTTGCTTTTTACTTA
V             AAAAAAAAAATGAAAAAAAATGAAAAAATGAAAAA-----AAAAATTTGCTTTTTACTTA
VI            AAAAAAAAAATGAAAAAAAATGAAAAAATGAAAAA-----AAAAATTTGCTTTTTACTTA
VII           AAAAAAAAAATGAAAAAAAATGAAAAAATGAAAAA-----AAAAATTTGCTTTTTACTTA
VIII          AAAAAAAAAATGAAAAAAAATGAAAAAATGAAAAA-----AAAAATTTGCTTTTTACTTA
IX            AAAAAAAAAATGAAAAAAAATGAAAAAATGAAAAA-----AAAAATTTGCTTTTTACTTA
X             AAAAAAAAAATGAAAAAAAATGAAAAAATGAAAAA-----AAAAATTTGCTTTTTACTTA
XI            AAAAAAAAAATGAAAAAAAATGAAAAAATGAAAAA-----AAAAATTTGCTTTTTACTTA
XII           AAAAAAAAAATGAAAAAAAATGAAAAAATGAAAAA-TGAAAAAAATTTGCTTTTTACTTA
XIII          AAAAAAAA--TGAAAAAAAATGAAAAAATGAAAAA-----AAAAATTTGCTTTTTACTTA
XIV           AAAAAAAAAATGAAAAAAAATGAAAAAATGAAAAA-----AAAAATTTGCTTTTTACTTA
XV            AAAAAAAAAAAAAAAAAAATGAAAAAAATGAAAAA---AAAAAAATTTGCTTTTTACTTA
XVI           AAAAAAAAAAAAAAAAAAATGAAAAAAATGAAAAA---AAAAAAATTTGCTTTTTACTTA
XVII          AAAAAAAAAATGAAAAAAATGAAAAAAATGAAAAATGAAAAAAAAAT-GCTTTTTACTTA
XVIII         AATGAAAAAATGAAAAAAAATGAAAAAATGAAAAAATGAAAAAAATTTGCTTTTTACTTA
Conservation  **  ****    *******   *************     ***** * ************

nr int                                                                     3060
I             CAAAAAAAGATCTAAGTTTAAATGAAGGA
II            CAAAAAAAGATCTAAGTTTAAATGAAGGA
III           CAAAAAAAGATCTAAGTTTAAATGAAGGA
IV            CAAAAAAAGATCTAAGTTTAAATGAAGGA
V             CAAAAAAAGATCTAAGTTTAAATGAAGGA
VI            CAAAAAAAGATCTAAGTTTAAATGAAGGA
VII           CAAAAAAAGATCTAAGTTTAAATGAAGGA
VIII          CAAAAAAAGATCTAAGTTTAAATGAAGGA
IX            CAAAAAAAGATCTAAGTTTAAATGAAGGA
X             CAAAAAAAGATCTAAGTTTAAATGAAGGA
XI            CAAAAAAAGATCTAAGTTTAAATGAAGGA
XII           CAAAAAAAGATCTAAGTTTAAATGAAGGA
XIII          CAAAAAAAGATCTAAGTTTAAATGAAGGA
XIV           CAAAAAAAGATCTAAGTTTAAATGAAGGA
XV            CAAAAAAAGATCTAAGTTTAAATGAAGGA
XVI           CAAAAAAAGATCTAAGTTTAAATGAAGGA
XVII          CAAAAAAAGATCTAAGTTTAAATGAAGGA
XVIII         CAAAAAAAGATCTAAGTTTAAATGAAGGA
Conservation  *****************************
```
