# Supplementary material for: Recombination in Glomus intraradices, a supposed ancient asexual arbuscular mycorrhizal fungus
Source: BMC Evol Biol. 2009 Jan 15;9:13. doi: 10.1186/1471-2148-9-13 (PMC2630297; doi:10.1186/1471-2148-9-13)

Additional file 4 - Phylogenetic relationships among genotypes in a population of *G. intraradices*

Neighbour-network using uncorrected *p* distance showing reticulate phylogenetic branching among a core set of genotypes. Bootstrap support of branches above 90% is indicated in % of 1000 replicates. Analyses were performed with two different restrictions. A. All indels were removed and only substitutions were considered in the analysis. B. Strongly divergent genotypes (XI, XV-XVIII) were removed from the analysis.

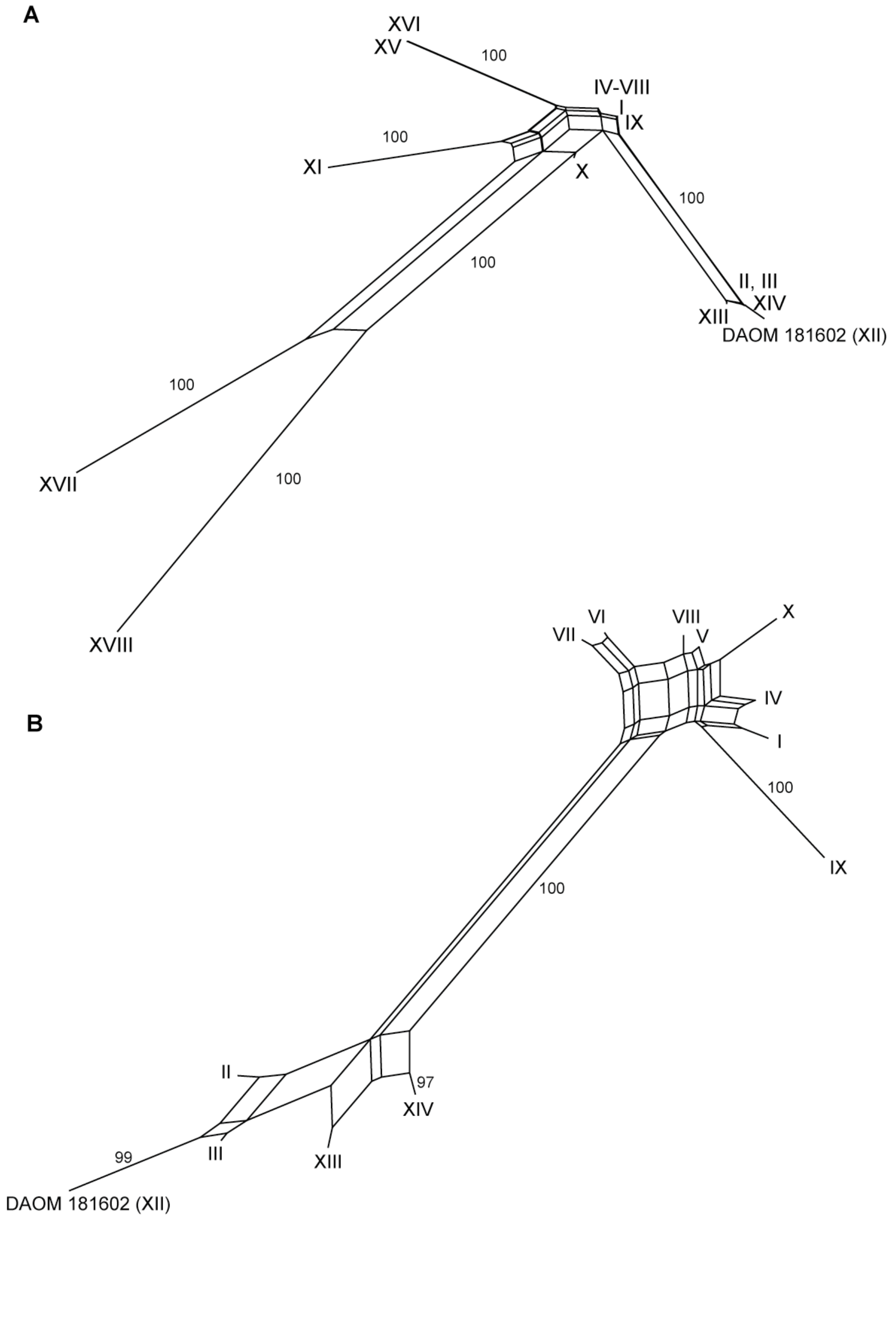

Supplement: Additional file 4 — Phylogenetic relationships among genotypes in a population of G. intraradices based on a Neighbour-network using uncorrected p distance. [file 1471-2148-9-13-S4.pdf]
