# Supplementary material for: Recombination in Glomus intraradices, a supposed ancient asexual arbuscular mycorrhizal fungus
Source: BMC Evol Biol. 2009 Jan 15;9:13. doi: 10.1186/1471-2148-9-13 (PMC2630297; doi:10.1186/1471-2148-9-13)

Additional file 5 – Summary of five recombination tests based on the concatenated sequences of 11 nuclear loci. Loci are concatenated arbitrarily according to the locus labels.

Colored shading indicates putative recombinant regions in the different sequences of the different genotypes. Different colors show results of the different tests. For individual tests, recombinant regions can overlap as all significant recombinant regions were kept in the analysis. Significance was based on $p < 0.05$, corrected for multiple comparisons.

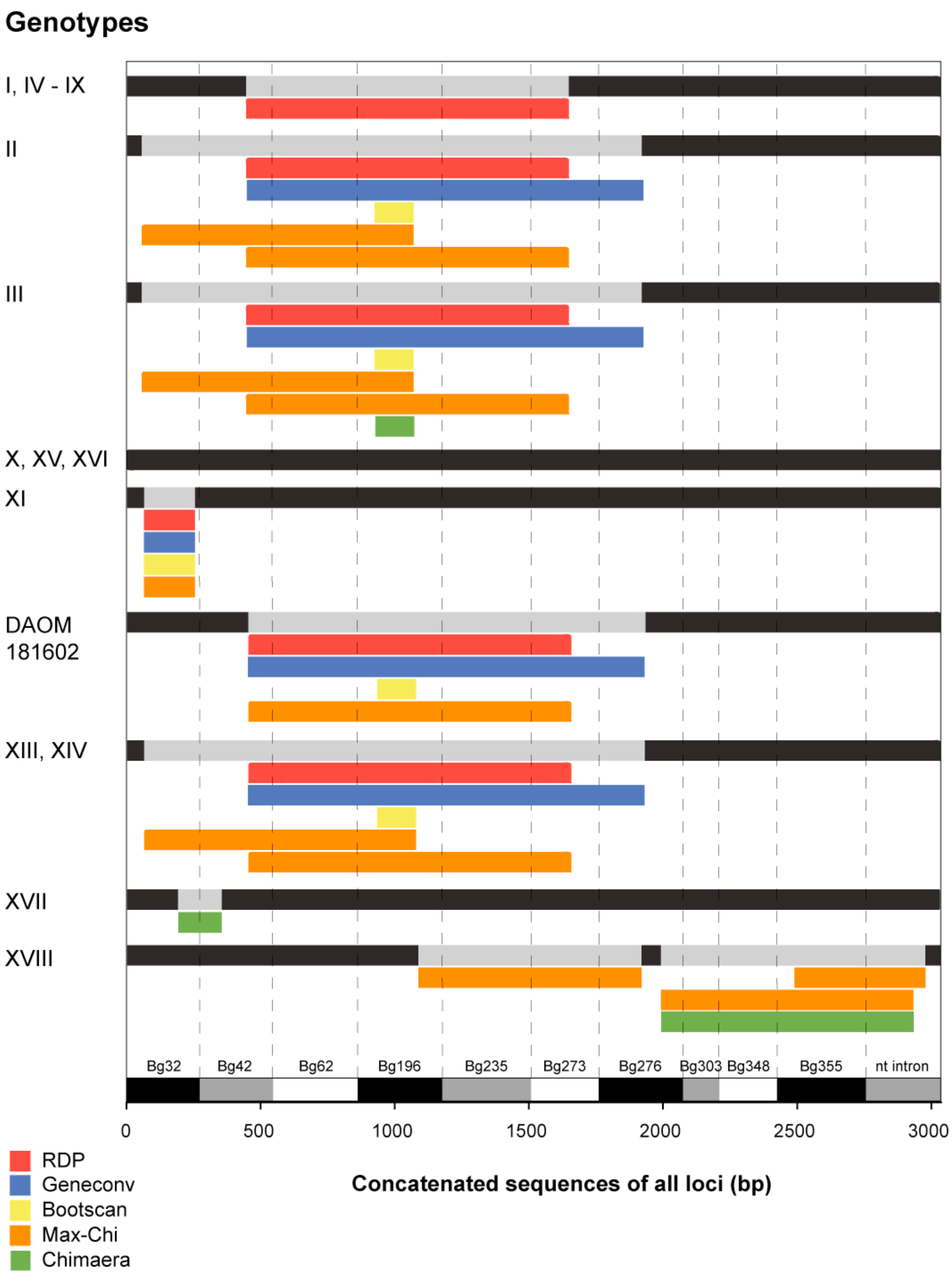

Supplement: Additional file 5 — Summary of five recombination tests based on the concatenated sequences of 11 nuclear loci. Loci are concatenated arbitrarily according to the locus labelling. For individual tests, recombinant regions can overlap as all significant recombinant regions were kept in the analysis. Significance was based on p < 0.05, corrected for multiple comparisons. For exact p values for all putative recombinant regions see additional file 2. The total alignment length is 3037 bp. [file 1471-2148-9-13-S5.pdf]
