# Supplementary material for: Recombination in Glomus intraradices, a supposed ancient asexual arbuscular mycorrhizal fungus
Source: BMC Evol Biol. 2009 Jan 15;9:13. doi: 10.1186/1471-2148-9-13 (PMC2630297; doi:10.1186/1471-2148-9-13)

Additional file 6 – Summary of five recombination tests based on the concatenated sequences of 11 nuclear loci. Loci Bg62, Bg196 and Bg235 showing strong signals of recombination in additional file 5 are not consecutive.

Colored shading indicates putative recombinant regions in the different sequences of the different genotypes. Different colors show results of the different tests. For individual tests, recombinant regions can overlap as all significant recombinant regions were kept in the analysis. Significance was based on $p < 0.05$, corrected for multiple comparisons.

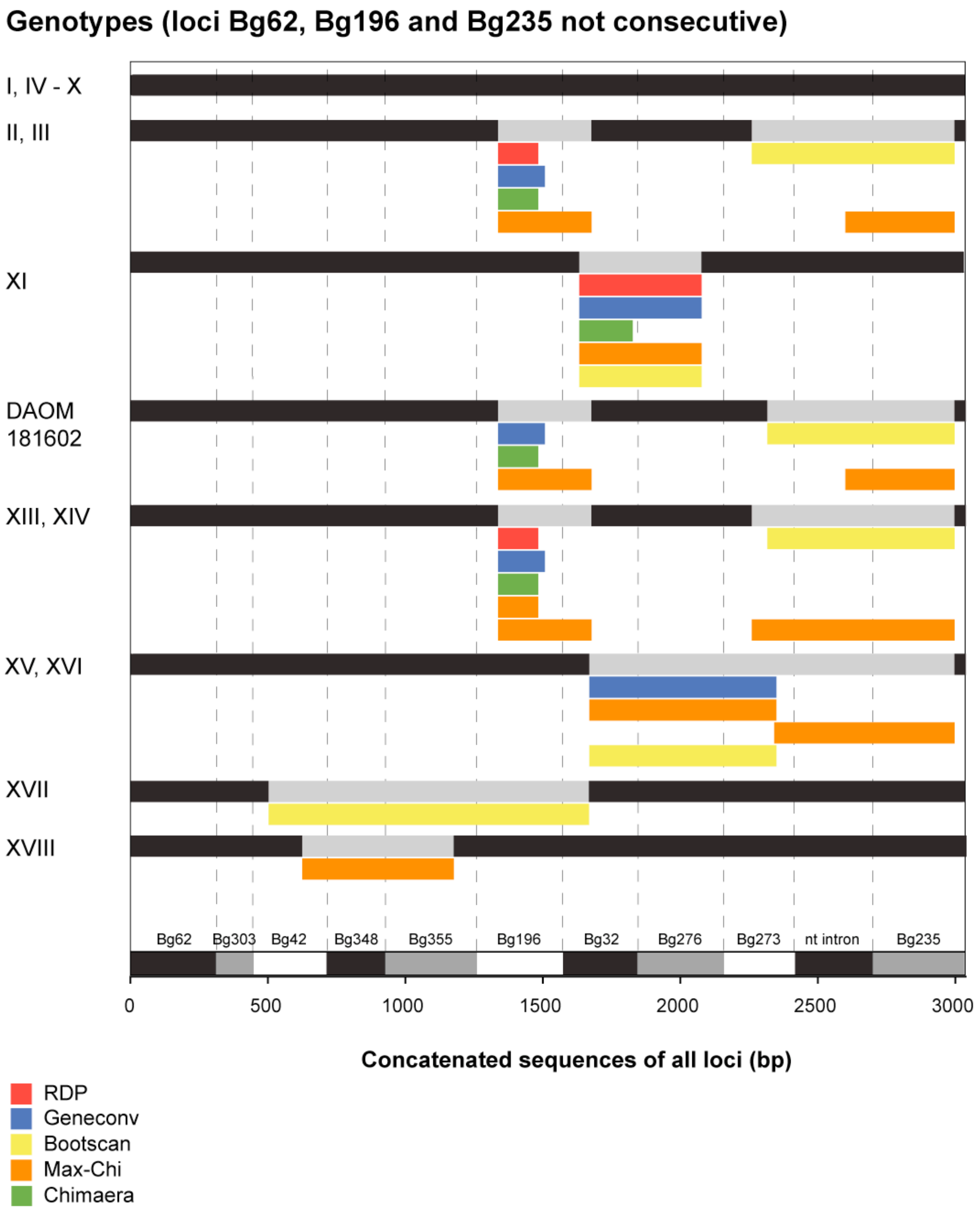

Supplement: Additional file 6 — Summary of five recombination tests based on the concatenated sequences of 11 nuclear loci. Loci Bg62, Bg196 and Bg235 showing strong signals of recombination in additional file 5 are not consecutive. [file 1471-2148-9-13-S6.pdf]
