# Supplementary material for: Recombination in Glomus intraradices, a supposed ancient asexual arbuscular mycorrhizal fungus
Source: BMC Evol Biol. 2009 Jan 15;9:13. doi: 10.1186/1471-2148-9-13 (PMC2630297; doi:10.1186/1471-2148-9-13)
Supplement: Additional file 8 — Details of the recombination breakpoint analysis with the Recombination Detection Program on concatenated sequences of the 11 loci among 17 genotypes of one field of Glomus intraradices and the isolate DAOM181602. The concatenation order corresponds to the one presented in additional file 5. [file 1471-2148-9-13-S8.pdf]

Additional file 8 – Details of the recombination breakpoint analysis with the Recombination Detection Program on concatenated sequences of the 11 loci among 17 genotypes of one field of *Glomus intraradices* and the isolate DAOM181602. The concatenation order corresponds to the arbitrary order presented in additional file 5.

Genotype	Breakpoints (bp)	Loci	Method	Probability
I, IV-IX	451-1657	Bg42-273	RDP	3.757E-02
II	451-1657	Bg42-273	RDP	1.536E-03
II	449-1921	Bg42-276	GC	1.939E-02
II	929-1067	Bg196	BOOT	1.999E-02
II	56-1067	Bg32-Bg196	MAX	4.163E-03
II	451-1657	Bg42-273	MAX	2.961E-05
III	451-1657	Bg42-273	RDP	1.536E-03
III	449-1921	Bg42-276	GC	1.939E-02
III	929-1067	Bg196	BOOT	2.268E-02
III	56-1067	Bg32-Bg196	MAX	4.079E-03
III	451-1657	Bg42-273	MAX	2.961E-05
III	929-1067	Bg196	CHIM	1.517E-02
XI	56-239	Bg32	RDP	1.300E-03
XI	56-239	Bg32	GC	1.134E-02
XI	56-239	Bg32	BOOT	1.084E-03
XI	56-239	Bg32	MAX	4.651E-03
DAOM181602	451-1657	Bg42-273	RDP	2.798E-03
DAOM181602	449-1921	Bg42-276	GC	3.610E-02
DAOM181602	929-1067	Bg196	BOOT	4.559E-02
DAOM181602	451-1657	Bg42-273	MAX	2.095E-04
XIII, XIV	451-1657	Bg42-273	RDP	1.536E-03
XIII, XIV	449-1921	Bg42-276	GC	1.939E-02
XIII, XIV	451-1657	Bg42-273	MAX	2.961E-05
XIII	929-1067	Bg196	BOOT	1.013E-02
XIII	56-1067	Bg32-Bg196	MAX	1.058E-04
XIV	929-1067	Bg196	BOOT	8.906E-03
XIV	56-1067	Bg32-Bg196	MAX	1.080E-04
XVII	182-336	Bg32-Bg42	CHIM	1.516E-03
XVIII	2002-2948	Bg276-nr int	GC	4.803E-02
XVIII	1091-1922	Bg196-276	MAX	7.509E-04
XVIII	2503-2995	Bg355-nr int	MAX	2.939E-02
XVIII	2002-2948	Bg276-nr int	MAX	8.414E-04
XVIII	2002-2948	Bg276-nr int	CHIM	8.461E-06

Breakpoints were located in the sequence alignment of all loci concatenated in the following order: Bg32, Bg42, Bg62, Bg196, Bg235, Bg273, Bg276, Bg303, Bg348, Bg355 and nuclear intron. The total alignment length was 3037bp. Recombination tests included Bootscan (BOOT), Chimaera (CHIM), Geneconv (GC), MaxChi (MAX) and Recombination Detection Program (RDP). Probabilities are multiple comparisons corrected and represent the global K-A probability (Geneconv), binomial probability (RDP), regional probability (MaxChi and Chimaera), respectively.
