# Supplementary material for: Recombination in Glomus intraradices, a supposed ancient asexual arbuscular mycorrhizal fungus
Source: BMC Evol Biol. 2009 Jan 15;9:13. doi: 10.1186/1471-2148-9-13 (PMC2630297; doi:10.1186/1471-2148-9-13)

Additional file 9 – Partition homogeneity test of 11 nuclear loci

Partition homogeneity test showing the actual summed tree length by maximum parsimony of the dataset compared to summed tree lengths of 1000 artificially created datasets through resampling. The actual summed tree length is significantly shorter than the artificial datasets ($p = 0.011$), indicating that homoplasy exists among distant loci. All indels were removed and only substitutions were considered in the analysis.

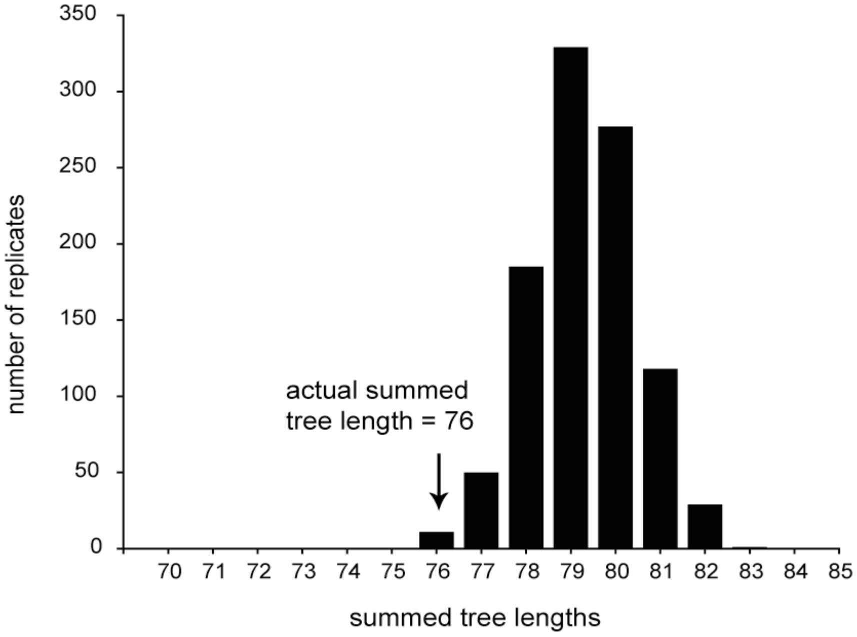

Supplement: Additional file 9 — Partition homogeneity test of 11 nuclear loci with all indels being removed. [file 1471-2148-9-13-S9.pdf]
